# Neutrophil extracellular traps mediate m^6^A modification and regulates sepsis-associated acute lung injury by activating ferroptosis in alveolar epithelial cells

**DOI:** 10.7150/ijbs.69141

**Published:** 2022-05-09

**Authors:** Hao Zhang, Jinlong Liu, Yilu Zhou, Mengdi Qu, Yanghanzhao Wang, Kefang Guo, Ruling Shen, Zhirong Sun, Juan P. Cata, Shuofei Yang, Wankun Chen, Changhong Miao

**Affiliations:** 1 Department of Anesthesiology, Zhongshan Hospital, Fudan University; Cancer Center, Zhongshan Hospital, Fudan University.; 2 Fudan Zhangjiang Institute, Shanghai 201203, China.; 3 Department of Anesthesiology, Shanghai Medical Colleague, Fudan University, 201203, Shanghai, China.; 4 Department of Anesthesiology, Shanghai Cancer Center, Shanghai, China.; 5 Department of Anesthesiology and Perioperative Medicine, The University of Texas MD Anderson Cancer Centre, Houston, TX, United States.; 6 Anesthesiology and Surgical Oncology Research Group, Houston, TX, United States.; 7 Shanghai Laboratory Animal Research Center, 201203, Shanghai, China.; 8 Department of Anesthesiology, Shanghai first Maternity and infant Hospital, Tongji University school of medicine, Shanghai, China.; 9 Department of Anesthesiology, Jinshan Hospital, Fudan University, Shanghai, China.; 10 Department of Vascular Surgery, Renji Hospital,School of Medicine, Shanghai Jiaotong University, Shanghai, China.; 11 Shanghai Key Laboratory of Perioperative Stress and Protection.

**Keywords:** Neutrophil extracellular traps, sepsis-associated acute lung injury, N6-Methylation, ferroptosis

## Abstract

Neutrophil extracellular traps (NETs) production is a major strategy employed by polymorphonuclear neutrophils (PMNs) to fight against microbes. NETs have been implicated in the pathogenesis of various lung injuries, although few studies have explored NETs in sepsis-associated acute lung injury (SI-ALI). Here, we demonstrate a major contribution of NETs to the pathology of sepsis-associated ALI by inducing ferroptosis of alveolar epithelial cells. Using both *in vitro* and *in vivo* studies, our findings show enhanced NETs accumulation in sepsis-associated ALI patients and mice, as well as the closely related upregulation of ferroptosis, the induction of which depends on METTL3-induced m6A modification of GPX4. Using a CLP-induced sepsis-associated ALI mouse model established with METTL3^-/-^ versus WT mice, in addition to METTL3 knockout and overexpression *in vitro*, we elucidated and confirmed the critical role of ferroptosis in NETs-induced ALI. These findings support a role for NETs-induced METTL3 modification and the subsequent induction of ferroptosis in the pathogenesis of sepsis-associated ALI.

## Introduction

Acute lung injury (ALI) and acute respiratory distress syndrome (ARDS) are common lung disorders in critically ill patients 1. In 2016, ALI/ARDS accounted for more than 10% of all intensive care unit (ICU) and 4% of all hospital admissions in 50 countries 2, 3. Currently, patients with ALI/ARDS have a poor prognosis, and their mortality remains high (35-45%) 4. Even for patients who survive ALI, the long-term quality of life is adversely affected. Because of the high prevalence and unacceptably high mortality rate, ALI/ARDS remains a medical crisis. Low tidal volume ventilation, fluid-conservative therapy and anti-inflammatory drugs are options to treat ALI/ARDS 5; however, none are clinically effective. Hence, the underlying causes of sepsis-induced ALI need to be further identified to guide the development of novel therapeutics.

Sepsis is a frequent cause of ALI/ARDS 1, 2. A dysregulated inflammatory response and endothelial dysfunction are hallmarks of sepsis and ALI/ARDS. Polymorphonuclear neutrophils (PMNs) and platelets play a key role in sepsis. After recruitment to the infectious site, PMNs attack microorganisms using three main strategies: degranulation, phagocytosis, and the release of neutrophil extracellular traps (NETs). NETs are webs of DNA material and antimicrobial proteins. Platelets also promote NETs formation, as shown in the blood of septic patients 6. Studies have indicated that NETs are harmful in sepsis. *In vivo* imaging revealed a NETs-platelet-thrombin interaction that promotes intravascular coagulation in sepsis-induced organ injury 7. However, the role of NETs and their underlying molecular mechanisms in the development of sepsis-induced ALI is poorly defined.

Ferroptosis is an iron-dependent form of regulated necrosis characterized by phospholipid peroxidation caused by reactive oxygen species (ROS) produced during iron-mediated Fenton reactions. Autophagic degradation of ferritin reportedly causes labile iron accumulation and promotes ferroptosis 8, 9. Recent studies have shown that ferroptosis actively participates in many pathophysiological processes, including lung injury. For example, large amounts of iron accumulation have been observed in oleic acid-induced lung injury 10, intestinal ischemia/reperfusion-induced ALI 11, seawater drowning-induced ALI 12, and lipopolysaccharide (LPS)-induced ALI 13. Interestingly, NETs and ferroptosis share similar intracellular signaling processes, and both conditions have been described in ALI. However, limited evidence is available on how NETs and ferroptosis are implicated in sepsis-induced ALI/ARDS.

Using patient samples and a mouse model of sepsis-induced ALI with or without NETs formation inhibited by PAD4 depletion or NETs degradation induced by anti-Ly6G/DNase I treatment, we demonstrate that NETs contribute to the pathology of sepsis-induced ALI by inducing ferroptosis in alveolar epithelial cells. The induction of ferroptosis appears to depend on methyltransferase-like 3 (METTL3), which can regulate RNA metabolism 14. Using METTL3 knockout mice and METTL3 knockout and overexpression mice *in vitro*, we further verified that METTL3-induced m6A modification of GPX4 plays a key role in NETs-mediated ferroptosis. We believe that blocking METTL3 can alleviate sepsis-induced ALI/ARDS.

## Methods

### Ethics Statement

This study was approved by the Ethics Committee of Fudan University Shanghai Cancer Center and updated in Zhongshan Hospital, Fudan University. The protocol in this study was performed in accordance with the principles of the Declaration of Helsinki. Written informed consent was obtained from patients or their relatives. All the mouse experiments were conducted according to the guidelines of the animal review committee at Zhongshan Hospital, Fudan University (protocol license number: 2020-119).

### Patients

Patients admitted to the intensive care unit (ICU) from January 2018 to December 2020 were enrolled in the study. Patients were included in the study if they were aged between 18 and 70 years, were diagnosed with sepsis or sepsis-induced ARDS after surgery and had an expected ICU hospitalization longer than 24 h. Exclusion criteria included a history of cardiopulmonary arrest before ICU admission, connective tissue disorders such as vasculitis, vascular embolism and pregnancy. Patients were diagnosed with sepsis according to the third international consensus definition for sepsis 15. ARDS was diagnosed according to the 2012 Berlin criteria 16.

### Data collection and blood sample measurements

Demographic data were recorded from patients following ICU admission. All the patients had undergone computed tomography (CT) to assess the severity of lung injury following ICU admission when the PaO_2_/FiO_2_ ratio was also calculated. Ten milliliters of peripheral venous blood was collected within 1 h of ICU admission. The peripheral blood neutrophil counts, lymphocyte counts, monocyte counts, platelet counts, hemoglobin concentration (g/dl), erythrocyte sedimentation rate, C-reactive protein levels, albumin concentration-activated partial thromboplastin time (in seconds), prothrombin time (in seconds), and fibrinogen and D-dimer concentrations were obtained and recorded. The interleukin 8 (IL-8) levels (pg/ml) were measured using an enzyme-linked immunoassay (ELISA) kit (ab229402; Abcam). Age-matched healthy subjects were used as controls (HCs) for laboratory studies. In these subjects, blood drawn was obtained in the morning during fasting conditions.

### Cecal ligation and perforation (CLP) mouse model

C57BL/6 mice (25-30 g, male) were purchased from Shanghai Laboratory Animal Research Center (Shanghai, China). Both male and female METTL3- or PAD4-knockout heterozygous mice were purchased from Shanghai Laboratory Animal Research Center (Shanghai, China). Next, 8- to 10-week-old mice were used to select age- and sex-matched WT (wild type) and METTL3 (METTL3^-/-^) or PAD4 (PAD4^-/-^)-KO mice for experiments and subject them to CLP under inhalational anesthesia with 2% isoflurane. Briefly, a 2-cm incision was made in the abdominal wall, and the cecum was exposed and ligated at a 0.5 cm point from the tip with 4-0 silk sutures. A 22-gauge needle was used to puncture through the distal cecum, extruding a small quantity of fecal content. The cecum was placed back into the abdominal cavity, and the exposed abdominal wall was closed in two layers using running 4-0 silk sutures. The development of sepsis-associated lung injury was determined as previously described 17. Mice allocated to the sham group only received cecal exposure without ligation or puncture. All the mice received 0.9% saline at the end of the surgery and analgesia and had free access to water and food.

To investigate the effect of NETs formation inhibition, depletion, and degradation on the progression of sepsis, we treated mice with CI-amidine, anti-Ly6G DNase I (Sigma Aldrich, USA) or MNase (Sigma Aldrich, USA) in 100 µl of 0.9% saline. DNase I can degrade NETs. Briefly, the mice were injected intravenously with 65 U of DNase I, MNase or DNase I combined with MNase every 2 days from cecal puncture to the end of the experiment or with 100 μl of 0.9% sodium chloride (control group). Anti-Ly6G depletes neutrophils at high doses 18. The mice were intravenously injected with anti-Ly6G (ab25377) (5 μg/g) one day before cecal puncture. Next, the mice were redosed at 2.5 μg/g every 2 days until the end of the experiment 19. Cl-amidine is an inhibitor of protein arginine deiminases (PADs). Mice in the Cl-amidine treatment groups were given doses of 50 mg/kg subcutaneously 20, 21 30-60 mins before CLP (control animals received PBS). The lung tissues were harvested and fixed in 4% paraformaldehyde at 4 °C or immediately stored at -80 °C. Whole blood was centrifuged at 3,000 rpm for 15 mins, and serum was collected and stored at -80 °C. Bronchoalveolar lavage fluid (BALF) collection was performed three times through a tracheal cannula with sterile PBS in a total volume of 1.5 mL and centrifuged at 1,500 rpm for 5 mins at 4 °C. To generate METTL3 overexpression, the mice were anesthetized with 5% isoflurane and AAV-6-METTL3 (obtained from Simo Biotech. Co., Ltd) was administered at a multiplicity of infection (MOI) dose of 10^6^ to the lung through an endotracheal tube inserted into the trachea. Three weeks later, the animals were used for *in vivo* studies. All the experimental procedures related to animals conformed to the guidelines from Directive 2010/63/EU of the European Parliament on the protection of animals used for scientific purposes or the NIH Guide for the Care and Use of Laboratory Animals. All the animals were euthanized with CO2 after the experiment.

### Quantification of cell-free DNA and MPO-DNA complexes

Cell-free DNA (cfDNA) in the serum and culture supernatant was detected using the Quant-It PicoGreen® dsDNA kit (Thermo Fisher) according to the manufacturer's instructions. The fluorescence intensity was measured at a 480 nm excitation wavelength and 530 nm emission wavelength to reflect the amount of DNA. Myeloperoxidase-DNA (MPO-DNA) complexes in the supernatant and plasma were detected using capture ELISA as previously described 22. Briefly, 75 μL of 5 μg/ml anti-MPO monoclonal antibody (ABD Serotec; Cat-No. 0400-0002), as the capturing antibody, was coated onto 96-well microtiter plates overnight at 4 °C. After blocking in 1% BSA, 40 μL of patient serum was added to the wells with peroxidase-labeled anti-DNA monoclonal antibody (component No. 2 of the commercial cell death detection ELISA kit; Roche, Cat. No. 11774425001). The plate was incubated for two hours at room temperature and washed three times with 200 μL of PBS per well. Next, the peroxidase substrate (ABTS) (Roche, Cat. No. 11774425001) was added. After 40 mins of incubation at 37 °C in the dark, the optical density (OD) was measured at 405 nm using a microplate reader.

### Human peripheral blood and mouse neutrophil isolation

Human neutrophils were isolated and purified as previously described 23. Briefly, blood was diluted with PBS and stratified on lymphocyte separation medium (commercially available biological product). After centrifugation at 800 g for 30 mins at room temperature, particles containing neutrophils and red blood cells (RBCs) were collected and resuspended in 3% dextran (spectrochemical), and the RBCs were precipitated at room temperature. After 30 mins, the supernatant was collected and centrifuged at 4 °C at 480 g for 5 mins. The remaining RBCs were separated from neutrophil granulocytes using BD Pharm Lyse Lysing Buffer (BD Biosciences). The resulting particles were then washed and resuspended in cold RPMI medium (Wisent Biologics) containing 3% fetal bovine serum (FBS). Neutrophil isolation and purification from mice were performed as previously described 24.

### NETs production and isolation

NETs production and isolation were conducted as previously described 25. Briefly, isolated human neutrophils were resuspended in RPMI 1640 (Thermo Fisher) and then seeded in 6-well plates (1.8×10^6^ cells per well). After stimulation with 50 nM phorbol 12-myristate 13-acetate (PMA) for four hours, the medium was removed, and the wells were washed with RPMI-1640 medium. At this concentration, PMA did not promote apoptosis or necrosis but caused the characteristic features of NETs formation. After removal of the supernatant, NETs adhered to the bottom were washed down by pipetting 2 ml of cold PBS and then were centrifuged at 1,000 g for 10 mins at 4 °C. After centrifugation at 20×g for 5 mins, the supernatant was obtained. The cell-free supernatant containing NETs (DNA-protein complex) was collected. The DNA concentration of NETs was measured by spectrophotometry using the Quant-it PicoGreen dsDNA reagent (Life Technologies), and NETs were used for further experiments 24, 26. The isolated NETs were either immediately used or snap frozen in liquid nitrogen.

### Immunofluorescence

NETs visualization was performed using immunofluorescence confocal microscopy, as previously described 27, 28. Patient samples were stained with anti-myeloperoxidase (diluted 1:200; Abcam 134132), anti-histone H3 (diluted 1:200, citrulline R2+R8+R17; Abcam, 5103), anti-neutrophil elastase (diluted 1:200; Abcam, 254178), a polyclonal goat anti-mouse Alexa Fluorite 647 antibody (Thermo Fisher) and anti-rabbit Alexa Fluorite 488 antibody (Thermo Fisher) as secondary Abs. The DNA was stained using DAPI (Sigma-Aldrich). For immunohistochemical studies, formalin-fixed and paraffin-embedded lung specimens from mice were stained with anti-citrullinated histone-3 (citH3, 1:200; Abcam) and anti-neutrophil elastase (diluted 1:100; Abcam, 254178). All the samples were observed under laser scanning confocal microscopy. Considering that myeloperoxidase (MPO) and neutrophil elastase (NE) are also located in neutrophils, we measured NETs specifically identified by positive staining using the anti-myeloperoxidase antibody and anti-citrullinated histone H3 antibody or positive staining with anti-neutrophil elastase and anti-citrullinated histone H3 29-31. The scoring criteria for NETs release from peripheral neutrophils were quantified by examining 100 neutrophils. The immunostaining images in serial sections from each case were estimated under high-power fields. All the sections were examined by at least two independent observers in a double-blinded manner and scored for further analysis and comparisons.

### Lung wet-to-dry ratio

The right lung was dissected, harvested, weighed (wet weight), and then dried overnight at 60 °C (dry weight). The wet weight/dry weight ratio was calculated by dividing the wet weight by the dry weight.

### Histological examination and immunohistochemistry

The right lung tissue was removed, washed with PBS, fixed with 4% formalin buffer solution (4% formaldehyde, 30 mM NaH_2_PO4, 45 mM Na_2_HPO_4_), and embedded in paraffin. For histological examination, formalin-fixed paraffin-embedded lung tissues were cut into 4-μm sections, placed on glass slides, dewaxed with xylene, and rehydrated with 30 to 100% ethanol. The sections were microwave-boiled in extract buffer (10 mM citric acid buffer, pH 6.0) for five minutes and stained with hematoxylin and eosin for histological examination. A semiquantitative scoring system was used to evaluate the severity of lung injury. The system evaluates four items, alveolar congestion and hemorrhage, alveolar wall/hyaline membrane formation, neutrophil infiltration or aggregation in alveoli or blood vessels, and the degree of inflammatory cell infiltration 32. Two pathologists blinded to the results scored the specimens as follows for each item: 0=no injury; 1=mild injury (25%); 2=moderate injury (50%); 3=severe injury (75%); 4=very severe injury (almost 100%). Next, each item was added for a possible maximum of 16 points (highest severity) 33.

For immunofluorescence staining, the sections were incubated at room temperature in blocking buffer (10% normal goat serum PBS, 0.1% Triton X-100) for 1 h and then incubated overnight at 4 °C with anti-GPX4 antibody (Abcam; ab125066; dilution 1:100). After washing, the anti-rabbit HRP secondary antibody (ab205718; Abcam) was incubated at room temperature for one hour, the avidin-biotin-peroxidase complex was incubated for 15 mins, and diaminobenzidine staining was performed for 5 mins. The specimens were analyzed under light microscopy (Carl Zeiss, Jena, Germany).

### Enzyme-linked immunosorbent assay (ELISA)

Mouse whole blood was collected into a syringe via heart puncture and was placed in a test tube at room temperature for 30 mins. After centrifugation (6,000 RPM; 15 mins; 4 °C), the plasma and supernatant were immediately frozen in liquid nitrogen and stored at -80 °C for further analysis. The concentrations of IL-1β, IL-8, TNF-α, TGF-β, ferritin, and glutathione (GSH) in plasma were measured by ELISA (Boster, China) according to the manufacturer's instructions.

### Global m6A measurement

The level of global m6A in total RNA of HPAEpiC cells treated with or without NETs was quantified using an EpiQuik m6A RNA Methylation Quantification Kit (Epigentek Group, Farmingdale, NY) as described previously 34, 35.

### m6A dot blot assay

The levels of m6A in HPAEpiC cells treated with or without NETs were measured according to qualitative m6A modifications as previously described 36. Briefly, poly-A RNA was purified using a Dynabeads mRNA Purification Kit (Thermo Fisher, Carlsbad, CA, USA) and spotted onto an Amersham Hybond-XL membrane. After incubation with the anti-m6A primary antibody and mouse-HRP secondary antibody, the membrane was incubated with Pierce ECL2 Western blotting substrate and exposed to X-ray Super RX Film.

### Iron assay

The content of ferrous iron (Fe^2+^) in HPAEpiC cells was determined using an iron assay kit (Abcam, USA). Samples were first collected, washed with cold PBS, and homogenized in iron measuring buffer, and then reductant was added to the collected supernatant for mixed incubation. Finally, an iron probe was added, mixed, and incubated for one hour, and the content was immediately determined by colorimetry (absorbance: 593 nm; OD: 593 nm).

### Malondialdehyde (MDA) quantification

A lipid peroxidation assay kit (Abcam) was used to measure the relative concentration of MDA in HPAEpiC cells. MDA-thiobarbituric acid (TBA) admixtures were formed by the MDA-TBA reaction between MDA and TBA in the samples and were quantified using the OD532 nm colorimetric method.

### Cell culture

Human alveolar epithelial (HPAEpiC) cells were obtained from ScienCell Research Laboratories (Carlsbad, CA) and cultured in Dulbecco's modified Eagle's medium (DMEM)/F12 (HyClone, Logan, UT) supplemented with 10% fetal bovine serum in a 5% CO_2_/95% air incubator. METTL3 knockout HPAEpiC cells were generated via the CRISPR/Cas9 method 37. Briefly, CRISPR/Cas9 technology was used to generate the METTL3^-/-^ HPAEpiC cell line. First, METTL3 sgRNA (5'-TCAGGAGTTGATTGAGGTAAAGCGAGGTCTAGG-3') was designed by http://crispr.mit.edu and inserted into the pLVX-sgRNA plasmid, and METTL3 sgRNA-containing lentivirus was generated in 293T cells. HPAEpiC cells were seeded into 10 cm tissue culture dishes and cotransfected with METTL3 sgRNA expression lentivirus and Cas9 expression lentivirus for 48 h. Next, HPAEpiC cells were trypsinized using 0.25% trypsin (Gibco) and seeded as single cells into 96-well plates. Cell clones with METTL3 knockout were identified via western blotting and Sanger sequencing.

### Cell viability

HPAEpiC cell viability after treatment with NETs was determined using the cell counting kit 8 (CCK-8) method as previously described 38. Briefly, the treated cells were collected, and the culture supernatant was converted to 10% CCK-8 fresh medium. The absorbance at 450 nm was determined using a multimode microplate reader.

### ROS Quantification

Intracellular ROS production in HPAEpiC cells was monitored using the fluorescent probe DCFH-DA and a reactive oxygen species assay (Beyotime, China). DCDF-DA is transformed into DCFH and fluorescent DCF by intracellular esterase and ROS, respectively. Briefly, HPAEpiC cells were incubated with DCFH-DA, washed with PBS, excited at 488 nm and emitted at 525 nm by flow cytometry analysis (Beckman Coulter, Inc.).

### Real-time PCR analysis

Real-time PCR was performed as previously described 39. Briefly, lung tissues or HPAEpiC cells were treated with TRIzol (Thermo Fisher, USA), and total RNA was extracted using an RNA extraction kit (TaKaRa, Japan). After reverse transcription of RNA into cDNA (Lianchuan Biology), PCR was performed to amplify the target gene. Changes in the expression levels were compared with those of GAPDH.

### Western Blotting

Western blotting was performed as described in our previous study 40. Briefly, protein was isolated from lung tissues or cells, separated by 12% SDS-PAGE, and transferred to PVDF membranes. The membranes were then probed with the following primary antibodies: anti-GPX4, anti-TLR9, anti-MyD88, anti-p65, anti-METTL3 (Abcam) and anti-GAPDH (Santa Cruz Biotechnology). The antibodies were incubated overnight at 4 °C and further visualized using enhanced western blot chemiluminescence detection reagents (Amersham Biosciences Inc., Piscataway, NJ, USA).

### Me-RIP assay

Methylated m6A RNA immunoprecipitation (me-RIP) was performed to analyze the methylated GPX4 mRNA level of HPAEpiC cells using an anti-m6A antibody (Abcam; ab151230; dilution, 1:500) as described previously 41. GO and KEGG analyses were performed as previously described 42.

### RNA-seq analysis

Total RNA was extracted using TRIzol reagent (TIANGEN, China) and detected on a 1% agarose gel. The purity, concentration, and integrity of the total RNA samples were assessed before further analysis. After cluster generation, the library preparations were sequenced on the Illumina HiSeqTM 4,000 platform by Biomarker Technologies (Beijing, China), and the raw reads were generated. Next, the raw reads were filtered by removing adapter and poly-N sequences and inferior-quality reads from the raw reads. The clean reads were mapped to the Rattus norvegicus (Rnor_6.0) reference genome sequence using HISAT2 tools. The gene expression levels were estimated by determining the fragments per kilobase of transcript per million fragments mapped. Gene expression analysis of the different groups was performed by DESeq2. Genes with a *P* < 0.05 were defined as differentially expressed genes (DEGs). Next, we used the KOBAS 3.0 platform to perform enrichment analysis of the DEGs. The KEGG plot and heatmap were generated using the OmicShare online tools (http://www.omicshare.com/tools). The raw data are available from the corresponding author upon reasonable request.

### Statistical Analysis

The data were expressed as means ± standard error of the means (SEM). For two-group comparison analysis, 2-tailed t test (unpaired) was used. For multiple comparisons, ANOVA followed by Tukey's correction was performed. *P* < 0.05 was considered statistically significant. GraphPad Prism version 9.0 software (GraphPad Software, Inc., La Jolla, CA, USA) was used for statistical analysis.

## Results

### Enhanced NETs levels indicate disease severity in sepsis-associated ALI patients

**Figure 1:**
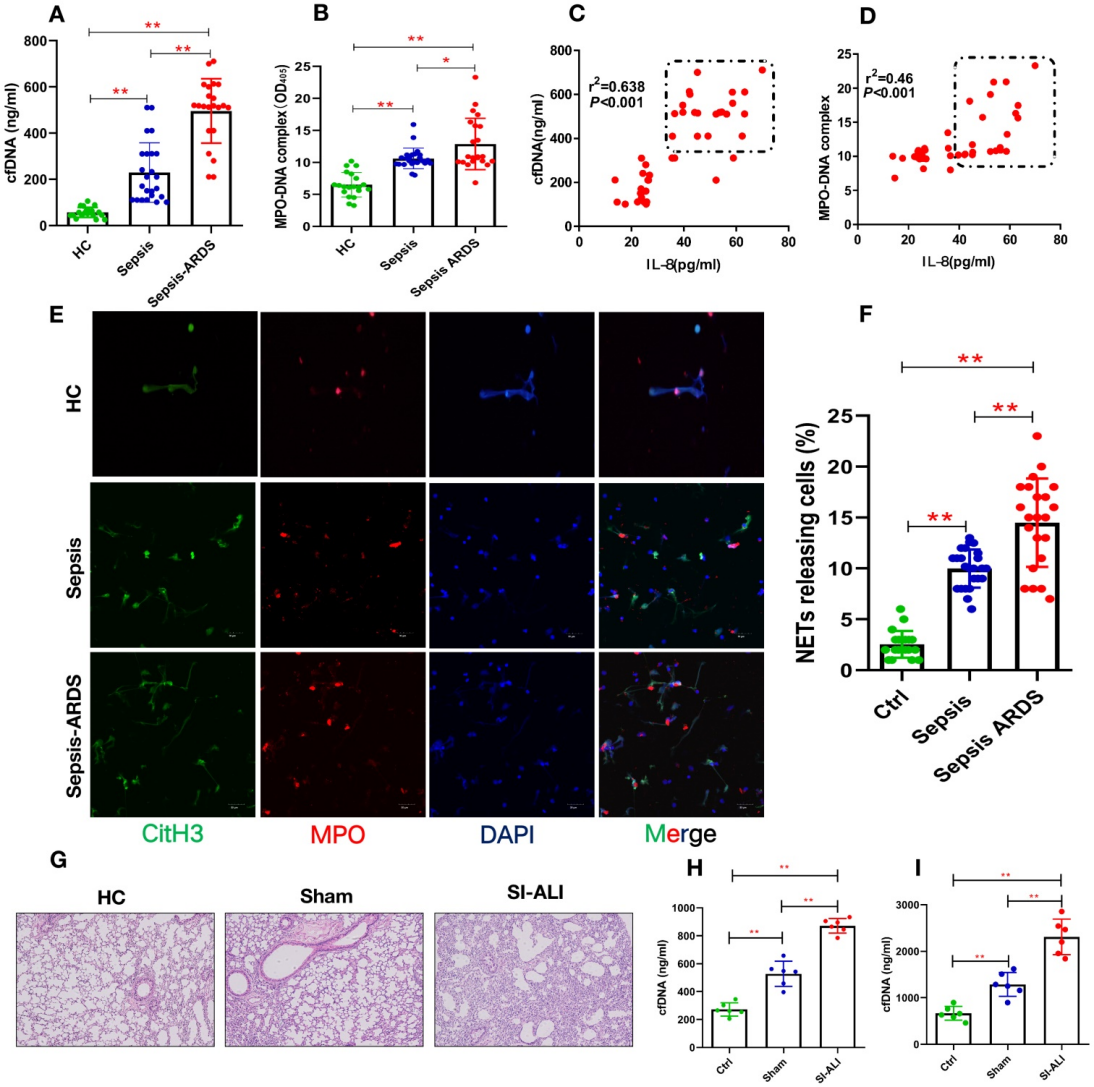
** Enhanced NETs release in sepsis-associated ALI patients and mouse models. (A)** Serum cell-free DNA and **(B)** MPO-DNA complexes were detected in healthy controls, sepsis patients, and patients with sepsis-induced ARDS. HC (n=20), Sepsis (n=24), Sepsis ARDS (n=22). **(C)** A positive correlation was found between serum cell-free DNA and **(D)** MPO-DNA complexes concerning the IL-8 level in healthy controls and patients with sepsis ARDS. HC, Sepsis ARDS; dot in box means Sepsis ARDS patients; **(E, F)** NETosis representative pictures in the HC group, Sepsis group and Sepsis ARDS group (red: MPO, green: CitH3, blue: DAPI). **(G)** Paraffin-embedded lung tissue samples were stained for H&E. Representative images of H&E staining are shown at 200× magnification showing lung injury in the HC group, sham group and sepsis ALI group in mice (n=6). **(H, I)** Cell-free DNA in plasma and BAL in mice of the HC group, sham group and sepsis group (n=6 in each group, BAL: bronchoalveolar lavage). *P<0.05, **P<0.01 (two-way ANOVA with Tukey's correction).

To elucidate the involvement of NETs in sepsis-associated ALI, we measured the levels of cfDNA and MPO-DNA in patients with sepsis and septic ARDS, as well as healthy controls. Compared with healthy controls, both septic patients and those with septic shock/ARDS showed increased cfDNA (**Fig. 1A**) and MPO-DNA (**Fig. 1B**) levels. Briefly, 3-fold and 4-fold changes were observed in sepsis patients and septic ARDS patients, respectively, suggesting an association between NETosis and clinical severity. The positive correlation of the serum cfDNA and MPO-DNA levels with IL-8 levels (**Fig. 1C-D**) further supports the role of enhanced NETosis levels in the development of sepsis-associated lung injury and is associated with diagnosis severity. The baseline characteristics of healthy controls and patients are shown in (**Supplementary Table 1**).

Higher NETs levels are usually associated with impaired NETs degradation. We used the CLP model to verify the role of NETs *in vivo*. After the CLP model was verified to be stable, it was used for future experiments (**Supplementary Fig. 1A, B**). The serum from ARDS murine sepsis mice showed a decreased NETs degradation ability compared with that from sham mice (**Supplementary Fig. 1C**) that was fully reversed by DNase I treatment alone and significantly improved by combined DNase I and MNase treatment (**Supplementary Fig. 1D,E**).

### NETs are increased in humans and mice with sepsis-associated ARDS

Significantly higher levels of NETs, indicated by coexpression of CitH3 and MPO in blood, were observed in humans with sepsis-associated ARDS (**Fig. 1E and Fig. 1F**), and enhanced CitH3-NE coexpression was observed in mouse lung tissue (**Supplementary Fig. 2A**). As expected, along with significant lung inflammation and lung injury (**Fig. 1G, Supplementary Fig. 2B**), the cfDNA levels in both the plasma and BALF from sepsis-associated ALI mice were increased by more than 3-fold compared with those in healthy controls and significantly higher than those in sham mice (**Fig. 1H-I**). Increased cfDNA levels in both the plasma and BALF were significantly reduced by DNase I treatment and even more robustly reduced by anti-Ly6G treatment (**Supplementary Fig. 2C and 2D**). Overall, our findings in patients and mice show that the accumulation of NETs plays a key role in the development of sepsis-associated lung injury and correlates with disease severity.

### Inhibiting NETs protects against lung inflammation in mice with sepsis-associated ALI

**Figure 2:**
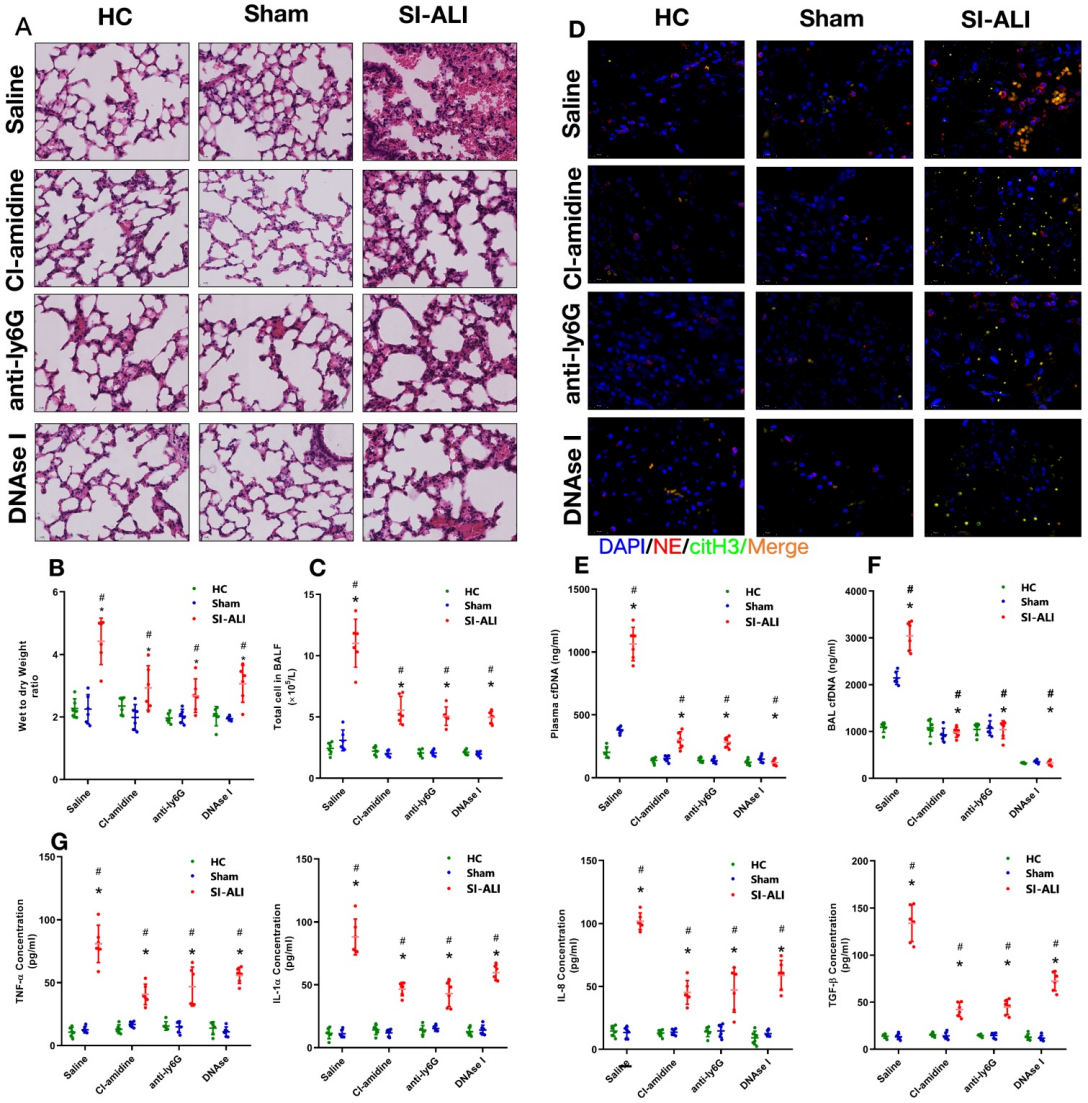
** NETs formation inhibition, NETs depletion or NETs degradation protects mice against sepsis-associated ALI.** A mouse model of cecal ligation and puncture (CLP)-induced sepsis-associated ARDS was established and then treated with saline, CI-amidine (inhibition of NETs formation), anti-ly6G (neutrophil depletion) or DNase (NETs degradation). **(A)** Paraffin-embedded mouse lung tissue samples were stained with H&E. Representative histological images are shown at 400× magnification. **(B)** Pulmonary edema was evaluated by determining the wet/dry weight ratio. **(C)** The cells in extracted bronchoalveolar lavage fluid (BALF) of mice were analyzed using a cell counter. **(D)** Representative images of immunofluorescence staining of NETs in lung tissues from HC, sham, and sepsis ARDS mouse models in the presence or absence of saline, CI-amidine, anti-ly6G and DNase (red: NE, green: CitH3, blue: DAPI). Scale bar=50 µm. **(E)** The cfDNA levels in plasma and **(F)** BALF were detected in the mouse model. **(G)** The levels of TNFα, IL-1α, IL-8 and TGF-β in plasma were determined by ELISA in a mouse model (n=6 in each group). *P<0.05. *indicates Sepsis-associated lung injury group versus HC group. # indicates Sepsis-associated lung injury group versus Sham group (Two-way ANOVA with Tukey's correction).

Additionally, we assessed whether NETs released during sepsis contributed to inflammation and lung injury. To test this hypothesis, we inhibited NETs formation or induced NETs degradation. Peptidylarginine deiminase type 4 (PAD4), the enzyme responsible for histone citrullination during NETosis and necessary for NETs formation, was inhibited by CI-amidine treatment. Anti-Ly6G treatment was administered to promote neutrophil depletion, and DNase I treatment was administered to promote NETs degradation. First, we showed massive infiltration of inflammatory cells in the lung tissues of sepsis-associated ALI mice compared with healthy and sham control animals. Cell infiltration was significantly reduced when NETs formation was inhibited by CI-amidine treatment, or NETs degradation was induced by anti-Ly6G and DNase I treatment (**Fig. 2A, Supplementary Fig. 2E**). Reduced inflammatory cell infiltration was accompanied by a reduction in the CitH3-NE complex (immunofluorescence characterization of NETs) in the lung tissue, as shown by confocal microscopy (**Fig. 2D**), indicating a reduced level of NETs formation after either CI-amidine, anti-Ly6G or DNase I treatment.

To evaluate lung damage and its associated inflammatory response, we calculated the wet-to-dry weight ratio, counted the total number of cells in BALF and measured the blood concentrations of inflammatory cytokines (TNF-α, IL-1α, IL-8 and TGF-β) after NETs inhibition. As expected, we observed a high wet-to-dry weight ratio in lung tissues, an increase in the total cell number in BALF and high serum concentrations of TNF-α, IL-1α, IL-8 and TGF-β in septic animals. These effects were significantly reduced by CI-amidine, anti-Ly6G or DNase I treatment (**Fig. 2B and 2C, 2G**) and accompanied by low levels of cfDNA in the plasma and BALF specimens (**Fig. 2E and 2F**). Collectively, these findings indicate that NETs play a role in the progression of sepsis-associated ARDS and that targeting NETs by inhibiting their formation or inducing their degradation is protective in mice with lung injury.

### NETs contribute to the progression of sepsis-associated ALI by inducing ferroptosis in alveolar epithelial cells

**Figure 3:**
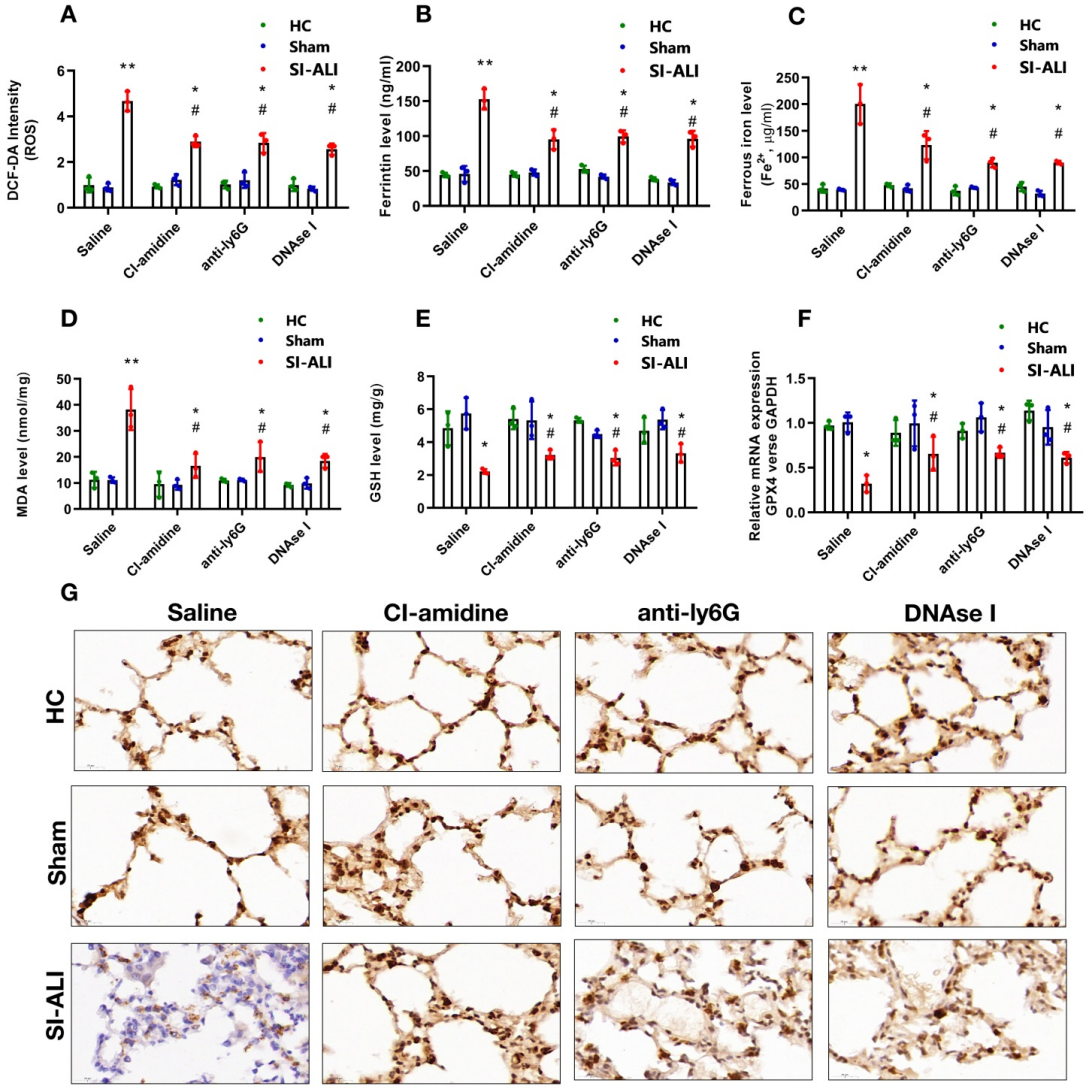
** NETs formation inhibition, NETs depletion and NETs degradation attenuate sepsis-induced ferroptosis in an ALI murine model.** A mouse model of CLP-induced sepsis-associated ALI was established and then treated with saline, CI-amidine (inhibition of NETs formation), anti-ly6G (neutrophil depletion) or DNase (NETs degradation). **(A)** The ROS level was analyzed using the DCF-DA assay in mouse lung tissue. **(B)** The ferritin level was evaluated by ELISA in mouse lung tissue. **(C)** The ferrous iron (Fe^2+^) level was evaluated using an iron assay kit in mouse lung tissue. **(D)** The MDA level was evaluated using a lipid peroxidation assay kit in mouse lung tissue. **(E)** The GSH level was evaluated by ELISA in mouse lung tissue. **(F)** The mRNA level of GPX4 was analyzed by qRT-PCR in mouse lung tissue. **(G)** The protein level of GPX4 was confirmed by IHC in mouse lung tissue (n=6 in each group). *P<0.05, **P<0.01. *indicates Sepsis-associated ALI group versus HC group. # indicates Sepsis-associated ALI group versus Sham group (Two-way ANOVA with Tukey's correction).

Ferritin, ROS, and labile iron levels were measured to elucidate the involvement of ferroptosis in NETs-induced sepsis-associated lung injury. The ROS levels were increased approximately 5-fold in mice with lung injury compared with those in healthy and sham controls; however, this increase was significantly decreased by CI-amidine, anti-Ly6G or DNase I treatment (**Fig. 3A**). A similar decrease was observed in serum ferritin and labile iron levels (**Fig. 3B and 3C**). We observed higher concentration of MDA in ALI mice with sepsis which was reduced after treatment with CI-amidine, anti-Ly6G or DNase I (**Fig. 3D**).

The repair enzyme glutathione-peroxidase 4 (GPX4) negatively regulates ferroptosis by directly reducing lipid hydroperoxidation. We measured the GSH and GPX4 levels in mice. Compared with healthy and sham controls, mice with septic lung injury showed lower serum GSH levels, which were increased following CI-amidine, anti-Ly6G or DNase treatment (**Fig. 3E**). Both GPX4 mRNA and protein levels were reduced in mice with sepsis compared with those in the control and sham groups. Remarkably, CI-amidine, anti-Ly6G or DNase treatments restored the levels of GPX4 (**Fig. 3F and 3G, Supplementary Fig. 3A**). We then verified ferroptosis in lung tissue by transmission electron microscopy (TEM) in sepsis lung injury model (**Supplementary Fig. 3B**).

**Figure 4:**
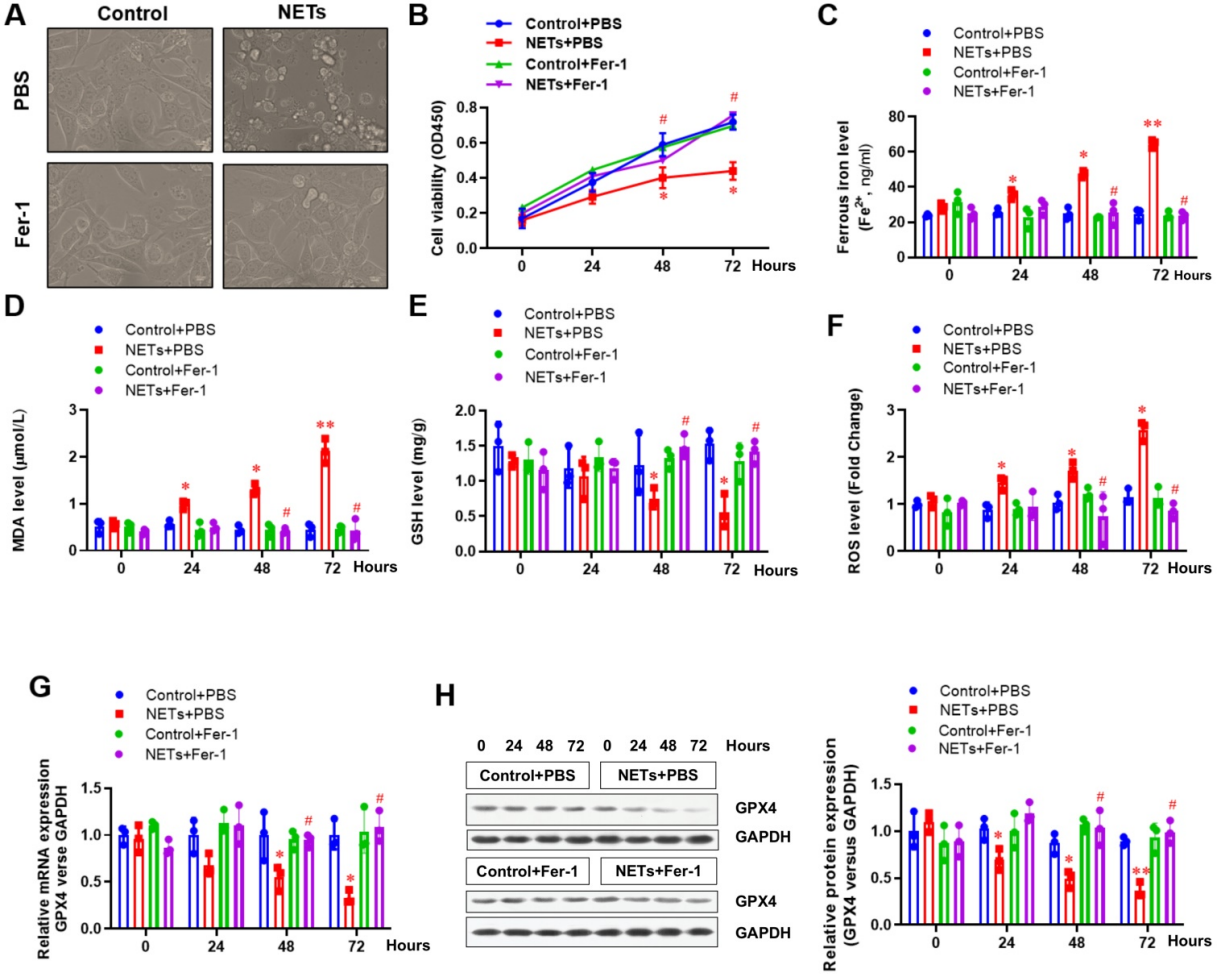
** NETs impair the viability of alveolar epithelial cells by inducing ferroptosis. (A)** Representative images of control and NETs-treated HPAEpiC cells with or without the ferroptosis inhibitor ferrostatin-1. (B) Cell viability was evaluated using the CCK-8 assay.** (C)** The ferrous iron (Fe^2+^) level was evaluated using an iron assay kit. **(D)** The MDA level was evaluated using a lipid peroxidation assay kit. **(E)** The GSH level was evaluated by ELISA. **(F)** The ROS level was analyzed using the DCF-DA assay. **(G)** The mRNA level of GPX4 was analyzed by qRT-PCR.** (H)** The protein level of GPX4 was analyzed by western blotting. *P<0.05, **P<0.01, #***P***<0.05. *indicates NETs+Fer-1 versus control+PBS. #indicates NETs+Fer-1 versus NETs +PBS (Two-way ANOVA with Tukey's correction).

Next, HPAEpiC cells were used to further confirm the involvement of ferroptosis in NETs-induced cell damage. Cells were treated with or without NETs for 48 h, and the morphology and other ferroptosis-related markers were detected to evaluate whether NETs treatment could induce ferroptosis. Using optical microscopy, we evaluated the viability of HPAEpiC cells treated with or without NETs. Compared with control cells, we observed decreased cell viability in NETs-treated HPAEpiC cells that was reversed by administering the ferroptosis inhibitor ferrostatin-1 (Fer-1) (**Fig. 4A**), indicating a potential role of ferroptosis in NETs-induced cell damage. Cell viability was then detected by CCK-8 assay. NETs treatment significantly decreased cell viability, which was reversed by Fer-1 (**Fig. 4B**). We further measured the levels of labile iron, MDA, GSH, ROS and GPX4 to confirm the induction of ferroptosis in NETs-treated HPAEpiC cells. Consistent with our observations *in vivo*, we observed a time-dependent increase in labile iron (**Fig. 4C**), MDA (**Fig. 4D**) and ROS (**Fig. 4F**) levels, which were all reversed by ferrostatin-1. Additionally, a time-dependent decrease in GSH (**Fig. 4E**) and GPX4 levels (**Fig. 4G and 4H**) was found following NETs treatment. As expected, the decreased GSH and GPX4 levels were reversed by further Fer-1 treatment (**Fig. 4E, 4G and 4H**). In summary, the data confirmed that ferroptosis was involved in NETs-induced alveolar epithelial cell damage and subsequent sepsis-associated ALI.

### NETs induce ferroptosis in alveolar epithelial cells by activating METTL3-mediated m6A modification

**Figure 5:**
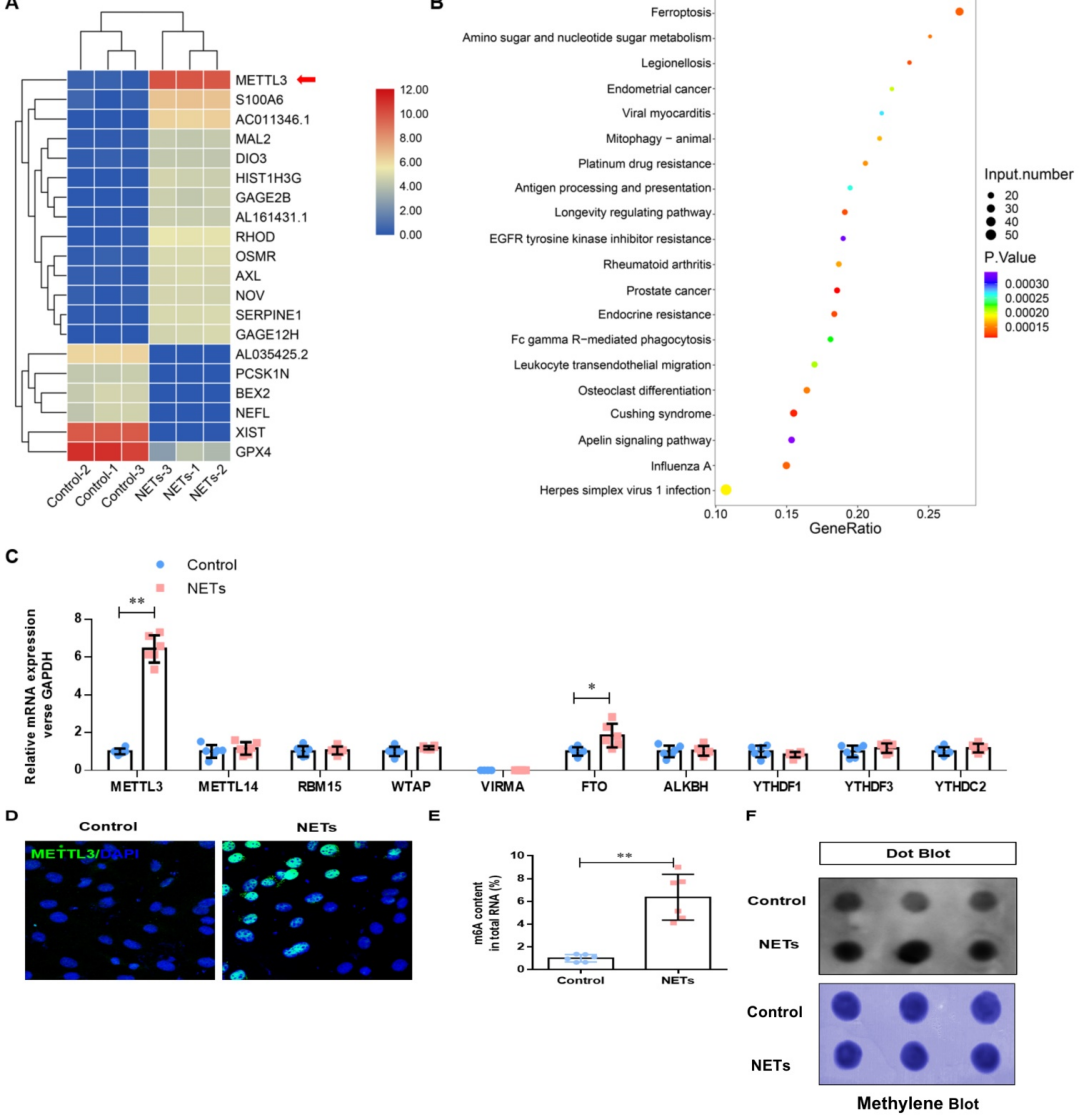
** NETs activate METTL3-mediated m6A modification in alveolar epithelial cells.** RNA-Seq identified METTL3 upregulation in NETs-treated alveolar epithelial cells. **(B)** GO and KEGG analyses. **(C)** m6A modification-related genes were confirmed by qRT-PCR. **(D)** The upregulated METTL3 protein in NETs-treated alveolar epithelial cells was confirmed by IF (green: METTL3, blue: DAPI). **(E)** The total level of m6A-methylated RNA was analyzed using a colorimetric kit and **(F)** the dot blot assay. *P<0.05, **P<0.01. (Two-way ANOVA with Tukey's correction).

To determine how NETs induce ferroptosis, we performed RNA-seq analysis in HPAEpiC cells treated with or without NETs. We found a 46.09-fold change in METTL3 upregulation in NETs-treated HPAEpiC cells (**Fig. 5A**). Additionally, Gene Ontology (GO) and KEGG pathway enrichment analyses demonstrated that ferroptosis signaling was activated in NETs-treated HPAEpiC cells (**Fig. 5B**). Next, we quantified the expression of the m6A modification-related genes and confirmed them by qRT-PCR. Both METTL3 and FTO were significantly upregulated in NETs-treated cells, with METTL3 expression increasing more than 12-fold (**Fig. 5C**). The upregulated expression of METTL3 in NETs-treated alveolar epithelial cells was further confirmed by immunofluorescence analysis (**Fig. 5D, Supplementary Fig. 3C**). METTL3 is a critical methyltransferase that catalyzes m6A methylation on mRNA or noncoding RNA. Thus, the total level of m6A-methylated RNA was detected using a colorimetric kit and the dot blot method. As expected, m6A-methylated RNA was significantly increased in NETs-treated cells compared with that in control cells, with a more than 12-fold increase (**Fig. 5E and 5F**).

### NETs promote METTL3 upregulation by activating the TLR9/MyD88/NF-kβ pathway in alveolar epithelial cells

**Figure 6:**
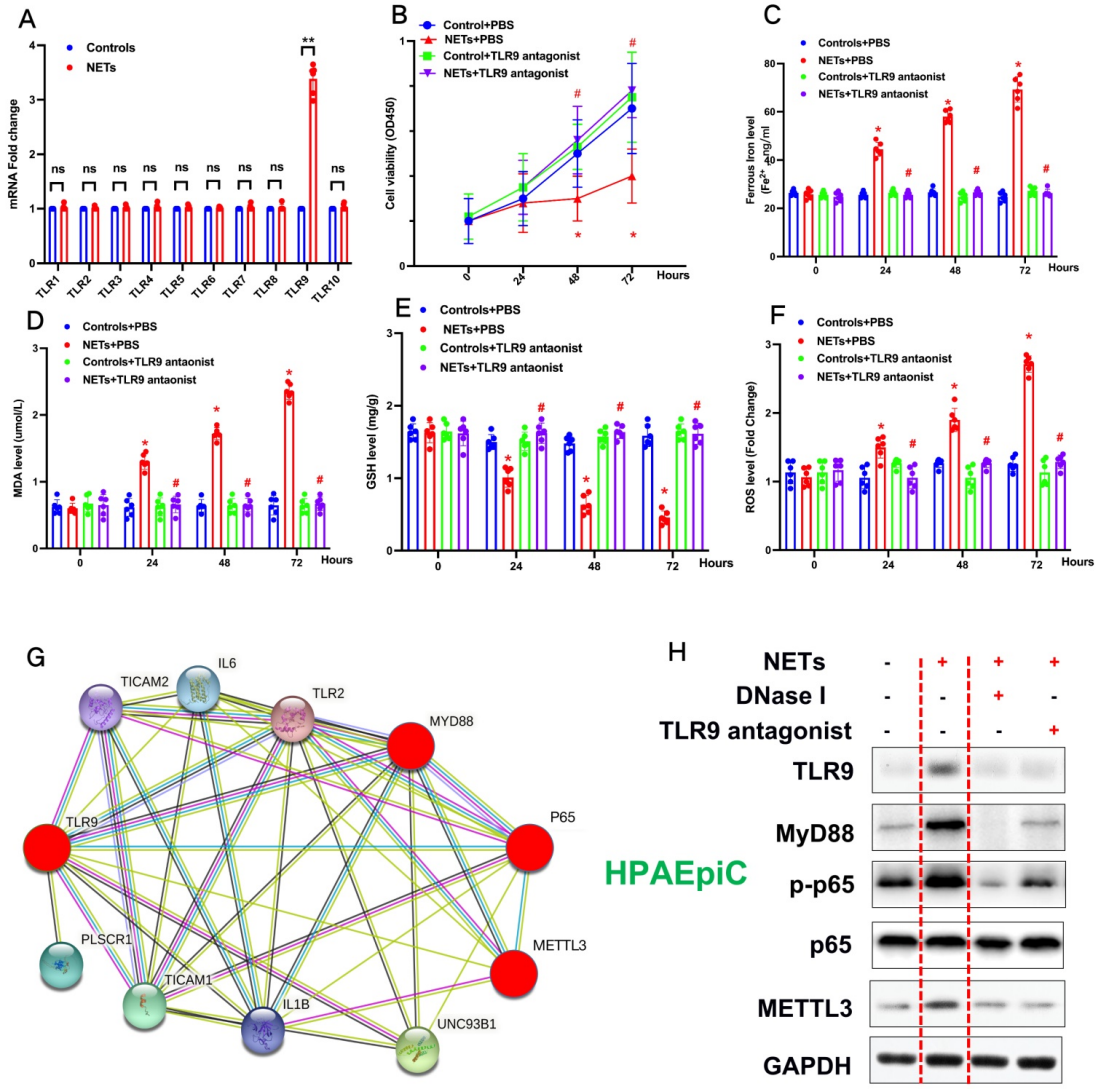
** NETs promote METTL3 upregulation via activation of the TLR9 pathway. (A)** qRT-PCR of TLRs at the mRNA level in alveolar epithelial cells treated with NETs or without NETs. **(B)** Cell viability was evaluated using the CCK-8 assay.** (C)** The ferrous iron (Fe^2+^) level was evaluated using an iron assay kit. **(D)** The MDA level was evaluated using a lipid peroxidation assay kit. **(E)** The GSH level was evaluated by ELISA. **(F)** The ROS level was analyzed using the DCF-DA assay. **(G)** Mev software of protein network predicting METTL3 upregulation via activation of the TLR9 pathway (http://string.embl.de/) **(H)** NETs significantly increased the expression of TLR9, METTL3, Myd88,p-p65 and METTL3 and the effects were abolished by adding DNAse I and TLR9 antagonist (2.5 µmol/L, ODNTTTGGG).

Previous studies have reported that NETosis results in the release of DAMPs, which induce an inflammatory reaction 2. We have reanalyzed our RNA-seq data and found that the Toll-like receptor pathway plays a vital role in NETs-induced upregulation of METTL3. TLRs are critical sensors of DAMPs, and mounting evidence has proven that TLR activation plays a significant role in sepsis lung injury progression 3. Therefore, in additional experiments, we evaluated the Toll-like receptor pathway (TLR1-TLR10) and verified that only the mRNA of TLR9 was significantly increased (**Fig. 6A**). Therefore, we evaluated the role of TLR9 in NETs-mediated cell viability. Compared with control cells, we observed decreased cell viability in NETs-treated HPAEpiC cells that was reversed by administering the TLR9 antagonist (2.5 mmol/L ODN-TTAGGG; InvivoGen) (**Fig. 6B**), indicating a potential role of TLR9 in NETs-induced cell damage. We further measured the levels of labile iron, MDA, GSH, and ROS to confirm the induction of ferroptosis in NETs-treated HPAEpiC cells. We observed a time-dependent increase in labile iron (**Fig. 6C**), MDA (**Fig. 6D**) and ROS (**Fig. 6F**) levels, which were all reversed by a TLR9 antagonist. Additionally, a time-dependent decrease in GSH levels (**Fig. 6E**) was found following NETs treatment. Mev software of protein network predicting NETs induced upregulation of METTL3 via TLR9/MyD88/NF-kβ pathway (**Fig. 6G**). In order to confirm the pathway, NETs inhibitor (DNase I) and TLR9 antagonist (2.5 µmol/L, ODNTTTGGG) were used to confirm. As expected, decreased TLR9, MyD88, p-p65 (NF-κB) and METTL3 levels were reversed by further DNase I and TLR9 antagonist treatment (**Fig. 6H**). In summary, the data confirmed that TLR9 was involved in NETs-induced alveolar epithelial cell damage and the subsequent METTL3 upregulation via TLR9/MyD88/NF-kβ pathway.

**Figure 7:**
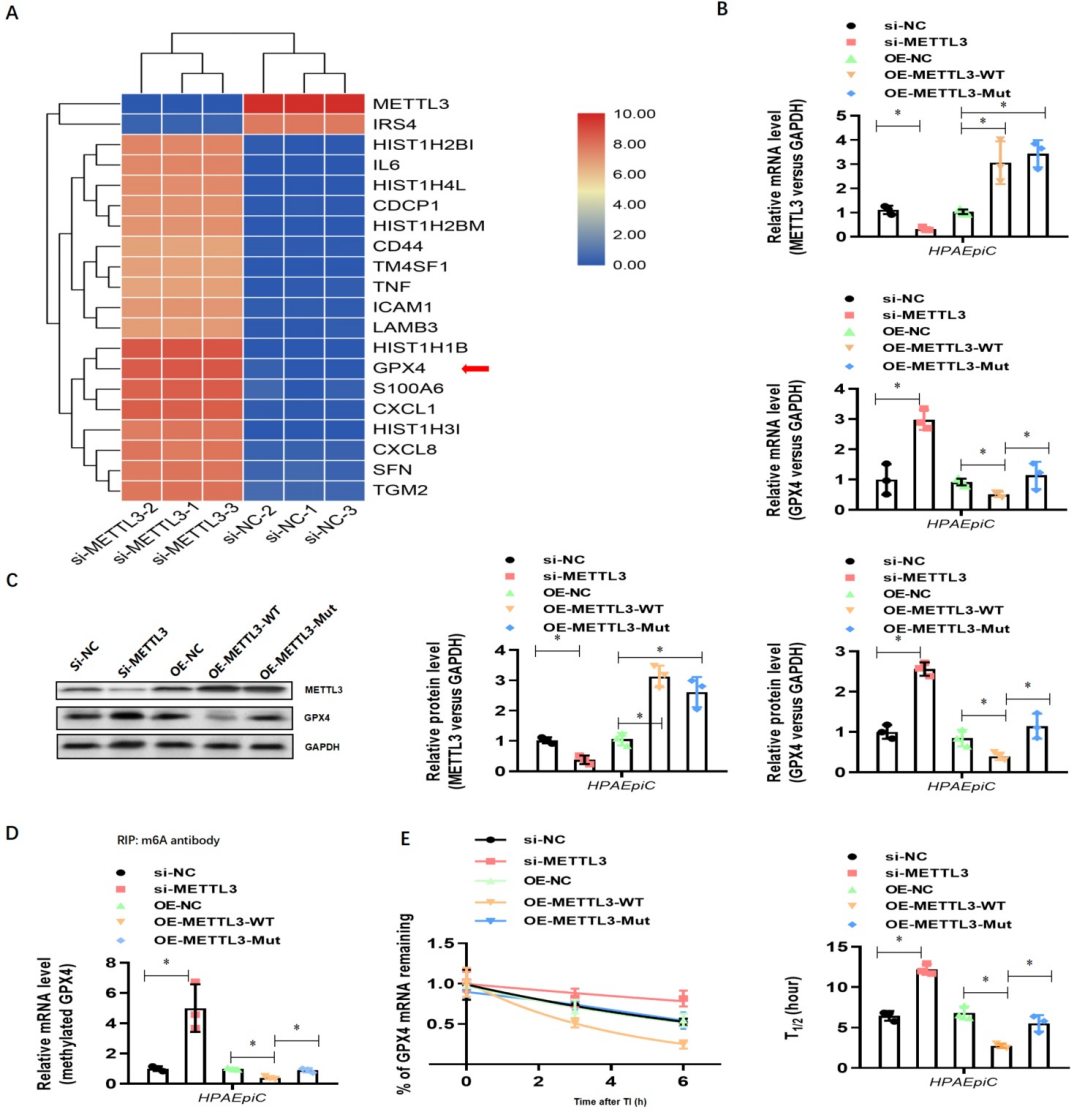
** NETs induced ferroptosis in alveolar epithelial cells. (A)** RNA-Seq+M6a-RIP-Seq identified GPX4 modification enrichment in alveolar epithelial cells. **(B)** METTL3 and GPX4 mRNA were analyzed by qRT-PCR.** (C)** METTL3 and GPX4 protein were analyzed by Western blotting.** (D)** m6A-methylated GPX4 mRNA was analyzed by Me-RIP-QPCR. **(E)** Curve and statistical analysis of GPX4 mRNA half-life after transfection with METTL3 siRNA, METTL3-WT, METTL3-Mut or the negative control after transcription inhibition (TI) are shown. *P<0.05 (two-way ANOVA with Tukey's correction).

To explore how METLL3 expression and its regulation of m6A methylation of RNAs regulate NETs-induced cell damage, RNA-seq plus m6A-RIP-seq was used to observe enriched m6A modifications on GPX4 genes (**Fig. 7A**). siRNA-mediated knockdown and lentivirus-mediated overexpression of METTL3 were performed, and efficient knockdown and robust overexpression were observed compared with those in controls using qPCR (**Fig. 7B**) and western blotting (**Fig. 7C**). We then measured the expression of both GPX4 and methylated GPX4 by qPCR or Me-RIP-qPCR and western blotting. Compared with the negative control groups (si-NC group cells), both higher GPX4 (**Fig. 7B and 7C**) and methylated GPX4 (**Fig. 7D**) were observed when METTL3 was knocked down in HPAEpiC cells (si-METTL3 group cells). By contrast, both GPX4 (**Fig. 7B and 7C**) and methylated GPX4 (**Fig. 7D**) were downregulated when METTL3 was overexpressed in OE-METTL3-WT cells compared with those in OE-NC and OE-METTL3-mut cells. The transcript inhibition assay further demonstrated that the half-life of GPX4 mRNA was markedly decreased with wild-type METTL3 overexpression but not with the catalytic METTL3 mutant (**Fig. 7E**). The total level of m6A-methylated RNA was detected using a colorimetric kit and the dot blot method. As expected, m6A-methylated RNA was significantly decreased in NETs-treated METTL3-/- cells compared with that in control cells (**Supplementary Fig. 4A**).

**Figure 8:**
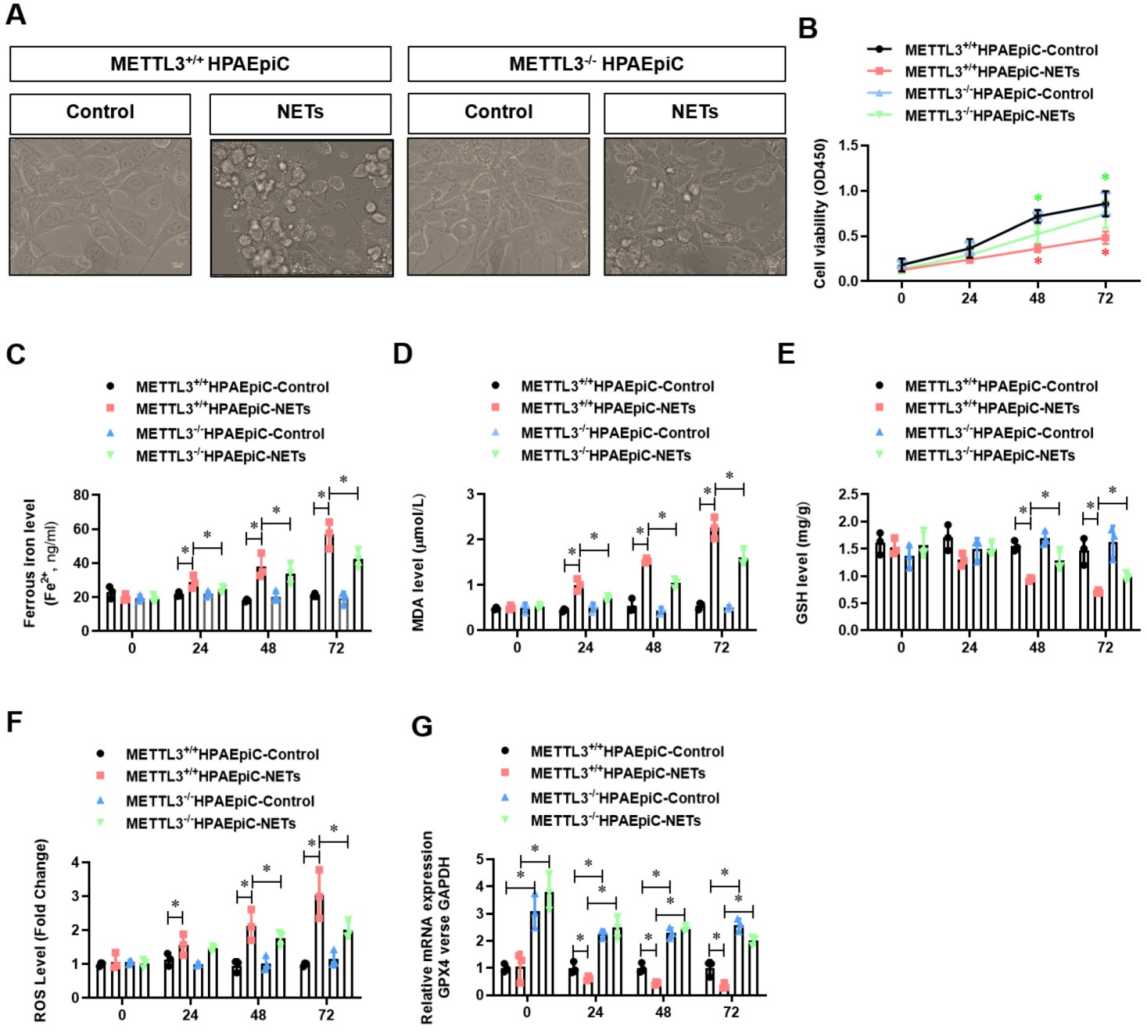
** METTL3 knockout attenuates NETs-induced ferroptosis in alveolar epithelial cells.** Representative images of control and NETs-treated HPAEpiC cells. **(B)** HPAEpiC cell viability was evaluated using CCK-8 assay (n=3); **(C)** ferrous iron (Fe^2+^) levels were evaluated using an iron assay kit in HPAEpiC cells (n=3);** (D)** MDA levels were evaluated using a lipid peroxidation assay kit in HPAEpiC cells (n=3); **(E)** GSH levels were evaluated by ELISA in HPAEpiC cells (n=3); **(F)** ROS levels were analyzed using the DCF-DA assay in HPAEpiC cells (n=3); **(G)** The mRNA levels of GPX4 were analyzed by qRT-PCR in HPAEpiC cells (n=3). *P<0.05. (Two-way ANOVA with Tukey's correction).

Because GPX4 is a key negative regulator of ferroptosis, we investigated whether METTL3 could affect ferroptosis. Thus, we first generated METTL3 knockout (METTL3^-/-^) HPAEpiC cells via the CRISPR/Cas9 method. Next, both METTL3^-/-^ and control METTL3^+/+^ cells were stimulated with NETs in culture for 24 h to induce ferroptosis. As expected, the morphology of control METTL3^+/+^HPAEpiC cells showed obvious shrinkage, which was significantly attenuated in METTL3^-/-^HPAEpiC cells (**Fig. 8A-B**). Next, the levels of labile iron, MDA, GSH and ROS were detected in the cultures at different time points. As expected, NETs induced higher labile iron (**Fig. 8C**), MDA (**Fig. 8D**), and ROS (**Fig. 8F**) levels but lower GSH (**Fig. 8E**) levels in the METTL3+/+HPAEpiC cell cultures than in the METTL3^-/-^HPAEpiC cell cultures. qPCR showed higher GPX4 expression at different time points in METTL3^-/-^HPAEpiC cells with or without NETs treatment (**Fig. 8G**), suggesting that METTL3 is a key negative regulator of GPX4 expression. In our study, GPX4 mRNA was degraded after m6A modification and then lead to ferroptosis of alveolar epithelial cells. We used Starbase2.0 database to predict the reader protein involved in GPX4mRNA degraded. Also, we have verify the level of YTHDF2 in alveolar epithelial cells treated with NETs or not. In our experiment, we found level of YTHDF2 in NETs treated group increased significantly compared with control group (**Supplementary Fig. 5A**), which is in parallel with the results from Starbase 2.0 (**Supplementary Fig. 5B-C**). All of these results together confirmed the m6A reader protein YTHDF2 mediated the GPX4mRNA degraded and then lead to ferroptosis of alveolar epithelial cells in sepsis acute lung injury. Overall, our findings demonstrated that NETs-induced ferroptosis in alveolar epithelial cells was dependent on METTL3-YTHDF2 mediated m6A modification of GPX4.

### METTL3 knockout inhibits NETs-induced ferroptosis in alveolar epithelial cells and protects mice against sepsis-associated ALI

**Figure 9:**
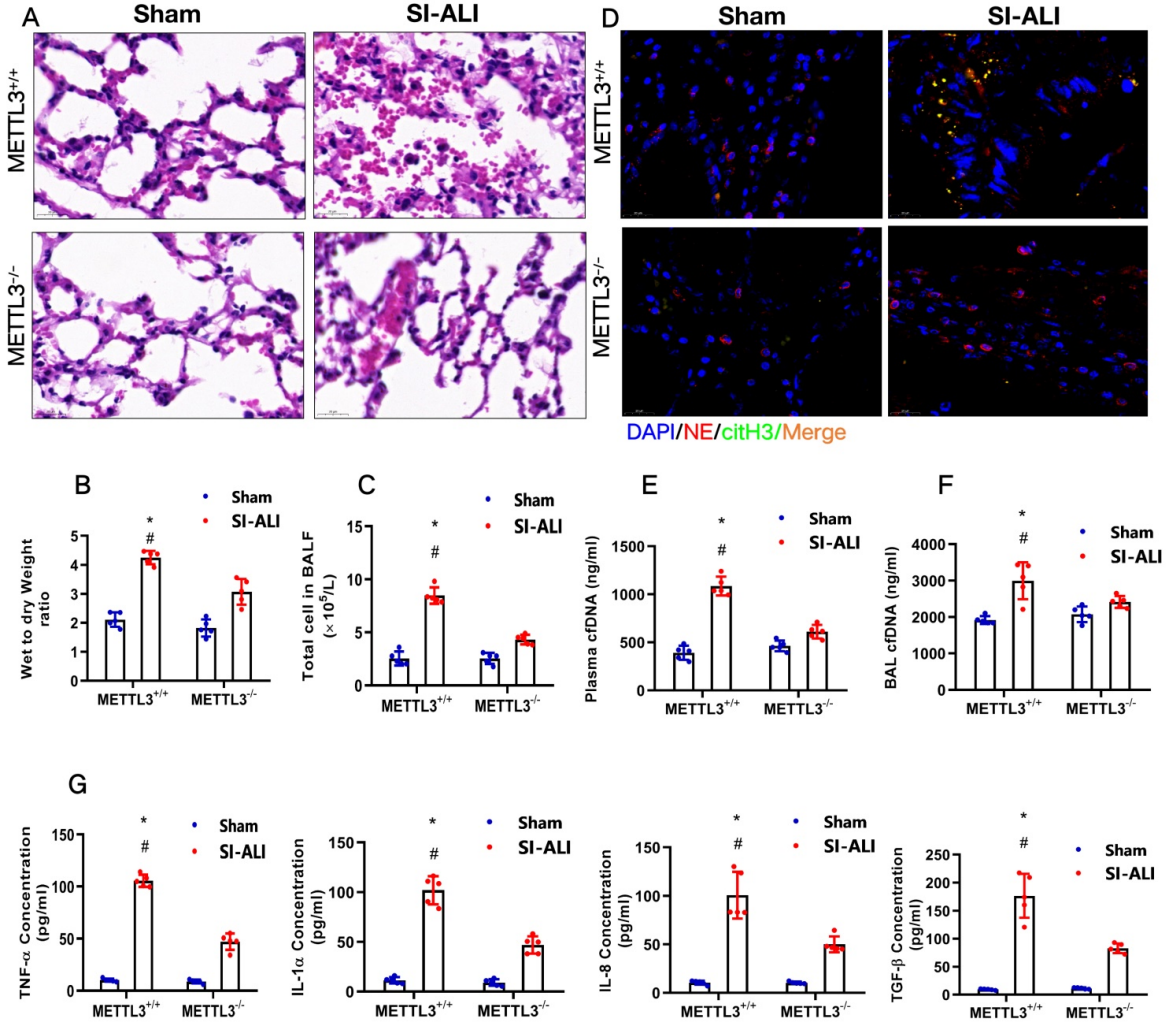
** METTL3 knockout protects mice against sepsis-associated ALI. (A)** Paraffin-embedded mouse lung tissue samples were stained with H&E. Representative histological images were shown at 400× magnification. Scale bar=50 µm. **(B)** Pulmonary edema was evaluated by determining the wet/dry weight ratio in a mouse model (n=6). **(C)** The cells in extracted BALF were analyzed by cell counting in a mouse model (n=6). **(D)** Representative images of immunofluorescence staining of NETs in lung tissues from METTL3^+/+^ and METTL3^-/-^ murine models in the sham and sepsis-associated lung injury group (red: NE, green: CitH3, blue: DAPI). **(E)** The cfDNA level in plasma (n=6) and **(F)** BALF was detected in METTL3^+/+^ and METTL3^-/-^ murine models in the sham and sepsis-associated lung injury groups (n=6). **(G)** The level of TNFα, IL-1α, IL-8 and TGF-β were determined by ELISA in METTL3^+/+^ and METTL3^-/-^ murine models in the sham and sepsis-associated lung injury groups (n=6). *P<0.05 (Two-way ANOVA with Tukey's correction).

To examine the role of METTL3 in sepsis-associated ALI *in vivo,* we used METTL3^-/-^ versus WT mice for CLP-induced sepsis-associated ALI. Compared with the sham group of mice, METTL3^+/+^ mice with sepsis-associated ALI showed massive infiltration of inflammatory cells in their lungs (**Fig. 9A**). The degree of lung damage in sepsis-associated ALI mice with sepsis with or without METTL3 expression was then evaluated by detecting the lung injury score, lung wet-to-dry weight ratio and total cells in BALF. As expected, compared with sham control mice, METTL3^+/+^ mice with sepsis-associated ALI showed a significant increase in the wet-to-dry weight ratio and total cell number in BALF that was reduced by METTL3 knockout (**Supplementary Fig. 6A, Fig. 9B and 9C**). Furthermore, systemic inflammation in METTL3^-/-^ mice was also reduced, as evidenced by lower serum concentrations of TNF-α, IL-1α, IL-8 and TGF-β (**Fig. 9G**). The reduced citH3-NE complex detected by IF analysis (**Fig. 9D**) and reduced cfDNA in both the plasma and BALF detected by ELISA (**Fig. 9E and 9F**) indicated that METTL3 knockout could significantly reduce NETs levels in sepsis-associated ALI mice. Lower NETs levels with METTL3 knockout were accompanied by reduced ferroptosis in sepsis-associated ARDS mice, as indicated by reduced serum levels of ROS (**Supplementary Fig. 6B**). We also found low levels of ferritin (**Supplementary Fig. 6C**) and labile iron (**Supplementary Fig. 6D**), reduced MDA and increased GSH concentrations (**Supplementary Fig. 6E and 6F**), and increased GPX4 expression (**Supplementary Fig. 6G and 6H**).

### PAD4 knockout attenuates sepsis-induced ferroptosis *in vivo* and protects mice against sepsis-associated ALI

**Figure 10:**
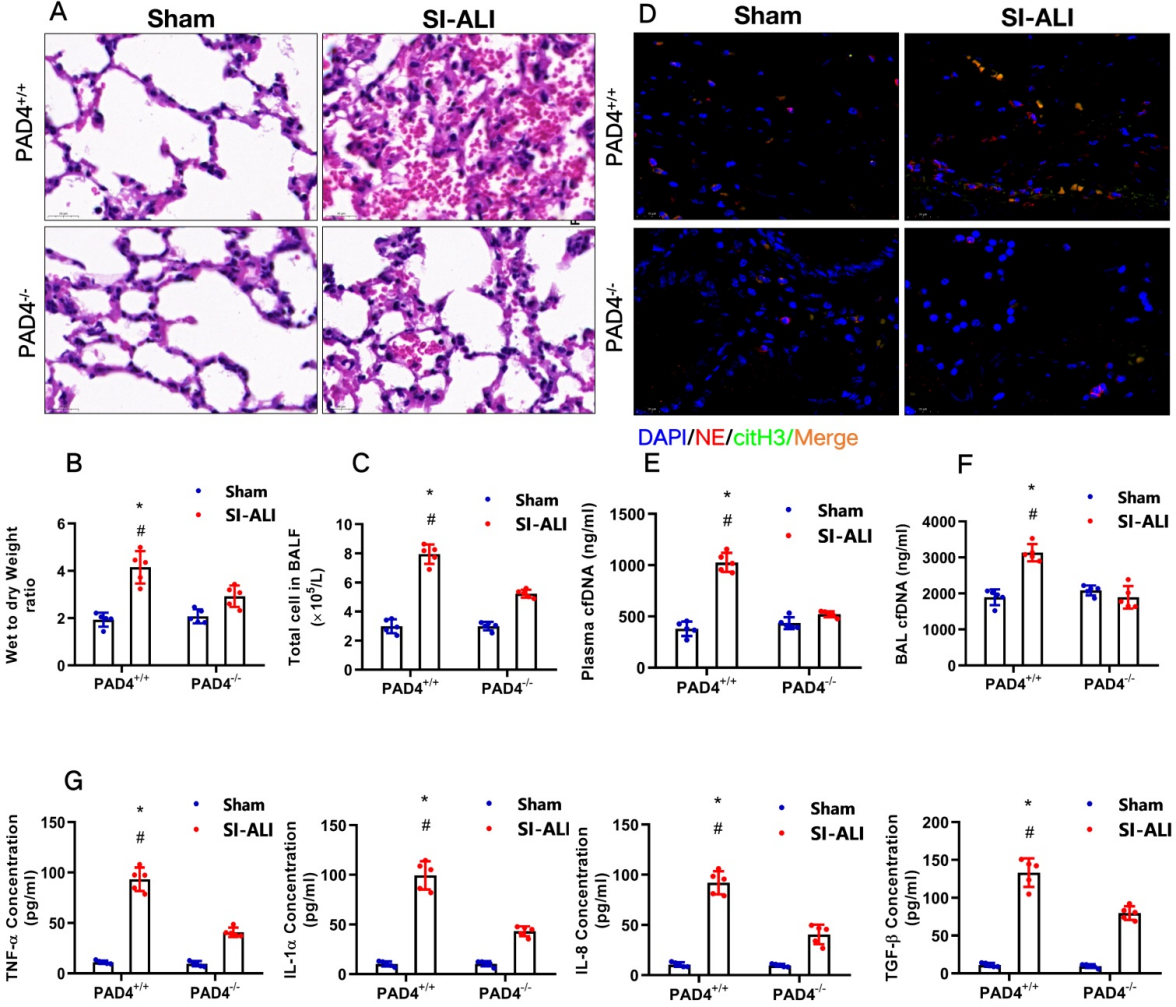
** PAD4 knockout protects mice against sepsis-associated ALI. (A)** Paraffin-embedded lung tissue samples were stained for H&E. Representative histological images are shown at 400× magnification. **(B)** Pulmonary edema was evaluated by determining the wet/dry weight ratio in PAD4^+/+^ and PAD4^-/-^ mouse models in the sham and sepsis-associated lung injury groups (n=6). **(C)** The cells in extracted BALF were analyzed by cell counting in PAD4^+/+^ and PAD4^-/-^ mouse models in the sham and sepsis-associated lung injury groups (n=6). **(D)** Representative images of immunofluorescence staining of NETs in lung tissues from the sham and sepsis-associated lung injury murine models. **(E)** The cfDNA levels in plasma (n=6) and **(F)** BALF were detected in PAD4^+/+^ and PAD4^-/-^ mouse models in the sham and sepsis-associated lung injury groups (n=6). **(G)** The levels of TNFα, IL-1α, IL-8 and TGF-β were determined by ELISA in PAD4^+/+^ and PAD4^-/-^ mouse models in the sham and sepsis-associated lung injury groups (n=6). *P<0.05, **P<0.01 (Two-way ANOVA with Tukey's correction).

Our experiments indicated that inhibiting NETs formation by reducing PAD4 activity could attenuate sepsis-induced ferroptosis and protect mice against sepsis-associated ALI. We then further confirmed the role of PAD4 in the development of sepsis-associated ALI by establishing CLP-induced sepsis-associated ALI using PAD4^-/-^ versus WT mice. Consistent with our observations, we found that using the PAD4 inhibitor CI-amidine reduced inflammation level in PAD4^+/+^ septic mice compared with that in sham mice (**Fig. 10A-C, Supplementary Fig. 7A**). Additionally, we observed significantly reduced inflammation and lung damage in PAD4^-/-^sepsis-associated ALI mice compared with those in PAD4^+/+^ sepsis-associated ALI mice, as indicated by significantly reduced inflammatory cell infiltration in the lung (**Fig. 10A**), a decreased wet-to-dry weight ratio of the lung (**Fig. 10B**) and a lower total cell number in BALF (**Fig. 10C**). As expected, systemic inflammation in PAD4^-/-^ mice was also reduced, as evidenced by lower serum TNF-α, IL-1α, IL-8 and TGF-β levels (**Fig. 10G**). The reduced citH3-NE complex detected by IF analysis (**Fig. 10D**) and lower cfDNA levels in both the plasma and BALF detected by ELISA (**Fig. 10E and 10F**) indicated that PAD knockout could significantly reduce NETs levels in sepsis-associated ARDS mice. The lower NETs level when PAD4 KO was accompanied by reduced ferroptosis in sepsis-associated ARDS mice, as demonstrated by reduced serum levels of ROS (**Supplementary Fig. 7B**), ferritin (**Supplementary Fig. 7C**) and labile iron (**Supplementary Fig. 7D**), reduced MDA and increased GSH levels in lung tissues (**Supplementary Fig. 7E and 7F**), and increased GPX4 expression detected by qPCR and IHC staining (**Supplementary Fig. 7G and 7H**). METTL3 expression was also decreased in PAD4^-/-^ mice compared with that in WT mice in the SI-ALI mouse model (**Supplementary Fig. 8A-B**). In summary, inhibition of NETs formation by depleting PAD4 expression attenuated sepsis-induced ferroptosis and protected mice against sepsis-associated lung injury.

## Discussion

In the present study, we demonstrated a major contribution of NETs to the pathology of sepsis-induced lung injury by inducing ferroptosis of alveolar epithelial cells. Elevated NETs and ferroptosis levels were detected in septic ARDS patients and SI-ALI mice. Blocking NETs formation through PAD4 depletion using both an inhibitor and PAD4^-/-^ mice and inducing NETs depletion or degradation by anti-Ly6G and DNase treatment significantly alleviated sepsis-induced ferroptosis and protected mice against SI-ALI. Furthermore, NETs-induced cell damage to human epithelial cells was reduced when ferroptosis was blocked. RNA-seq and qPCR analysis demonstrated that METTL3 was significantly elevated in NETs-induced ferroptosis of alveolar epithelial cells. Our *in vitro* model using METTL3 knockout and overexpression cells clarified the involvement of METTL3-induced m6A modification of GPX4 in NETs-induced ferroptosis of human alveolar epithelial cells. Our *in vivo* CLP-induced sepsis and SI-ALI mouse model using METTL3 KO mice further demonstrated their critical roles in NETs-induced cell ferroptosis and protecting mice against sepsis-associated lung injury. Taken together, these findings suggest that NETs-induced upregulation of METTL3 in alveolar epithelial cells and subsequent induction of ferroptosis play a novel role in sepsis-associated lung injury pathogenesis.

Sepsis-induced lung injury is a life-threatening condition with a high incidence and mortality rate^46^. In our study, enhanced NETs levels were associated with disease severity in sepsis-associated ALI patients and an SI-ALI mouse model. NETs contribute to the progression of ALI by direct or indirect lung damage, including disruption of lung endothelial and epithelial barriers 47. During NETosis, neutrophils produce and release NETs, which comprise extracellular deoxyribonucleic acid coated with histones, proteases and granular and cytosolic proteins, act as danger-associated molecular patterns and are associated with inflammation and tissue injury 48. Our previous study reported that tissue factor-enriched NETs promote immune thrombosis and disease progression in sepsis-induced lung injury 49. As DAMPs, NETs also induce damage and inflammatory responses in several pathological scenarios in different organs by opposing the NETosis of neutrophils 50. Among the proinflammatory cytokines, TNF-α, IL-1β, IL-6, and IL-8 are the most promising potential biomarkers to predict the morbidity and mortality of ALI/ARDS patients 51. In our study, the concentrations of proinflammatory cytokines were also increased in the CLP mouse model, a finding consistent with other study findings 52.

Additionally, we confirmed the role of NETs formation in the progression of lung injury. We inhibited NETs formation with inhibitors and found that inhibiting NETs formation alleviated lung inflammation in mice with SI-ALI. Notably, DNase I, a NETs degradation agent, demonstrated protective effects both *in vivo* and *in vitro* 53, 54. PAD4 is an essential enzyme for NETs formation 54. In our study, as an inhibitor of PAD4, Cl-amidine treatment decreased the concentrations of proinflammatory cytokines in a mouse model. Shi et al. reported NETs formation inhibition by Cl-amidine in colitis and colitis-associated cancer in mice, a finding consistent with our results 19. Additionally, we found that neutrophil depletion with anti-Ly6G decreased the concentrations of proinflammatory cytokines and the level of lung injury. Neutrophils play an essential immunoregulatory role in the inflammatory status of septic ALI 55. Although appropriate neutrophils from the circulation into the lungs are critical to clear pathogens from the alveolar space, excessive and persistent sequestration of neutrophils may cause injury to the lungs by releasing ROS and proinflammatory cytokines, exacerbating lung injury 55-58. Additionally, lung injury and the levels of proinflammatory cytokines were decreased in PAD4^-/-^ mice, indicating that a lack of the enzyme is required for chromatin decondensation to promote NETs release. In this regard, targeting neutrophils and NETs with inhibitors or degradation might be a potential therapeutic strategy for sepsis-associated lung injury that blocks the negative effects of neutrophils without altering their beneficial functions.

TLR9 pathway is important sensors of damage-associated molecular patterns (DAMPs) and mediated cellular interaction between immune cells and host cells in sepsis 19. TLR9 pathway activation also represent inflammatory phenotype 17. NETs contain DAMPs that could recognized by TLR9 31. TLR9 signal pathway mediated inflammatory effects of NETs on host cells 3,5. Previous studies has been reported that NETs could upregulated TLR9 expression in sepsis and sepsis lung injury 3,21,27. Also, TLRs is an upstream regulator of MyD88 and NF-kB which both involved in sepsis lung injury 6,7. In our further study, when blocking TLR9, we have found NETs failed to induce alveolar epithelial cells ferroptosis. Also, METTL3 expression decreased when DNAse I and TLR9 inhibitor added. These findings indicated that TLR9/MyD88/NF-kB as the major signaling in NETs-triggered METTL3 upregulation.

m6A is the most common and conserved internal modification in mammalian mRNA^59^. m6A modification has recently attracted attention because of its key role in gene expression regulation and human diseases 60-62. In the present study, we identified that m6A levels are significantly upregulated in NETs-treated alveolar epithelial cells *in vitro*. By modulating METTL3 expression through knockdown or overexpression in alveolar epithelial cells, we demonstrated that METTL3 is a key contributor to m6A levels. Previous studies have revealed that m6A modification can exert either oncogenic or tumor-suppressive effects in different ways 63, 64. In our study, in the METTL3^-/-^ SI-ALI mouse model, lung injury and the levels of proinflammatory cytokines were decreased compared with those in the METTL3+/+ SI-ALI mouse model, possibly leading to reduced infiltration of NETs in lung tissue. Simon et al. also demonstrated that decreased inflammation could inhibit NETosis 65. However, these studies focused on the role of METTL3 in mediating ferroptosis in alveolar epithelial cells rather than neutrophils. We showed that decreasing m6A levels through METTL3 silencing suppresses ferroptosis *in vivo* and *in vitro*. Fundamentally, we revealed that METTL3 mediates dynamic m6A modification on GPX4 and promotes ferroptosis of alveolar epithelial cells. Although no small-molecule inhibitors of METTL3 are currently available, inhibitors targeting demethylases (ALKBH5 and FTO) have been developed. Identifying small molecule inhibitors targeting METTL3 may provide an innovative treatment strategy for sepsis-induced lung injury.

Ferroptosis is a newly recognized type of cell death that is different from traditional necrosis, apoptosis, or autophagic cell death ferroptosis mainly results from iron-dependent lipid peroxidation, is closely related to glutathione peroxidase-4 (GPX4) inhibition and ROS accumulation and is characterized by mitochondrial shrinkage 46. In our study, indicators of ferroptosis in METTL3^+/+^ alveolar epithelial cells treated with NETs were increased compared with those in METTL3^-/-^ alveolar epithelial cells. Additionally, when a NETs inhibitor or NETs degradation was used in the SI-ALI mouse model, the indicators of ferroptosis were decreased compared with those in the control group, suggesting that NETs mediate ferroptosis in alveolar epithelial cells. Dong et al. reported that ferroptosis of alveolar epithelial cells was inhibited by nuclear factor E2 related factor 2 (Nrf2) by regulating SLC7A11 and HO-1, leading to alleviation of intestinal ischemia/reperfusion-induced ALI 67. Using inhibitors targeting ferroptosis may provide an innovative treatment strategy for sepsis-induced lung injury.

Our study has notable strengths. First, we integrated our data from humans, mice and cells to translate the role of NETs in SI-ALI. Additionally, we used multiple methods of NETs manipulation *in vivo* and *in vitro*. Second, RNA-seq was used to detect the role of METTL3-mediated m6A modification in NETs-mediated ferroptosis in sepsis-associated lung injury. Our study also has limitations. First, we used an anti-Ly6G clone (RB6-8C5) that is not specific to neutrophils because it also recognizes and depletes eosinophils. We solved this issue using pure neutrophils when isolated. Additionally, no data have quantified neutrophils in the lungs, BALF or blood. Second, we did not investigate NETs-induced ferroptosis of alveolar epithelial cells at the human subject level. Third, we did not correlate our findings in humans with clinical prognosis.

In conclusion, our findings support the role of METTL3 in sepsis-induced ALI pathogenesis through NETs-mediated m6A modification and subsequent ferroptosis of alveolar epithelial cells. We believe that this study provides critical clues to develop novel treatments for sepsis-induced ALI by targeting the PAD4/NETs/METTL3 pathway of alveolar epithelial cells.

## Supplementary Material

Supplementary methods, figures and tables.
