# Supplementary material for: Neutrophil extracellular traps mediate m6A modification and regulates sepsis-associated acute lung injury by activating ferroptosis in alveolar epithelial cells
Source: Int J Biol Sci. 2022 May 9;18(8):3337–57. doi: 10.7150/ijbs.69141 (PMC9134924; doi:10.7150/ijbs.69141)
Supplement: Supplementary file 1 — Supplementary methods, figures and tables. [file ijbsv18p3337s1.pdf]

Neutrophil extracellular traps mediates m^{6}A modification regulates sepsis-associated acute lung injury via activating ferroptosis of alveolar epithelial cells

Supplementary methods

Cecal ligation and perforation (CLP) mouse model stability

In order to confirm the stability of cecal ligation and perforation mouse model stability.Mortality and serum inflammatory was record in the experiments。Briefly,the mortality of mice within 72 hours after CLP surgery and serum level of IL-6, TNF-a, IL-10 were also tested with ELISA kit according to instructions (Abcam) in the experiments. Ten mice were used in each experiment in both sham or SI-ALI(CLP) group. These experiments were used to verify the stability of the CLP operation and to determine the number of samples needed to detect a series of indicators,[1-4]. After the CLP model is verified stability and been used for future experiment.

Transmission electron microscope(TEM)

We perform Transmission electron microscope(TEM) to detect ferroptosis in sepsis induced lung injury lung epithelial cells(5-6).Briefly,the lung tissues received a 2-h fixation in 0.05 M sodium cacodylate buffer with 2.5% glutaraldehyde at a pH of 7.2 at 25 °C. Next, they were incubated in 0.1 M sodium cacodylate buffer with 2% OsO_4 for 2 h and, then, in 1% aqueous uranyl acetate for 18 h. Following sequential ethanol-induced dehydration, the specimens were embedded in Epon 812 and cut into ultrathin sections using copper grids and stained with uranyl acetate and lead citrate before visualization under a Tecnai G2 spirit BioTwin transmission electron microscope (FEI Company, Hillsboro, Oregon).

Quantitative reverse transcription PCR

Quantitative reverse transcription PCR (qRT-PCR) was performed with a bio-RAD 96 instrument with the superreal premix following the manufacturers' instruction.All reactions were done in a 20 μL reaction volume in triplicate. Primers were obtained from Invitrogen. Following an initial denaturation at 95°C for 15 min, 40 cycles of PCR amplification were performed at 95°C for 10 s and 60°C for 20 s. Standard curves were generated and the fold change of target gene mRNA was normalized to β-actin. Melting

curve analysis was adopted to assess the specificity of amplification.The primer sequence is as below:

Gene	Forward	Reverse
**TLR1**	CCACGTTCCTAAAGACCTATCCC	CCAAGTGCTTGAGGTTCACAG
**TLR2**	ATCCTCCAATCAGGCTTCTCT	GGACAGGTCAAGGCTTTTTACA
**TLR3**	TTGCCTTGTATCTACTTTTGGGG	TCAACACTGTTATGTTTGTGGGT
**TLR4**	AGACCTGTCCCTGAACCCTAT	CGATGGACTTCTAAACCAGCCA
**TLR5**	CGATGGACTTCTAAACCAGCCA	GGTGAGGTTGCAGAAACGATAAA
**TLR6**	TTCTCCGACGGAAATGAATTTGC	CAGCGGTAGGTCTTTTGGAAC
**TLR7**	TCCTTGGGGCTAGATGGTTTC	TCCACGATCACATGGTTCTTTG
**TLR8**	ATGTTCCTTCAGTCGTCAATGC	TTGCTGCACTCTGCAATAACT
**TLR9**	CTGCCTTCCTACCCTGTGAG	GGA TGCGGTTGGAGGACAA
**TLR10**	AGGTTTGAGTGGGGCAAAAA T	CCATCACGCAAAAGAACCCAG

Gene	Forward primer (5'-3')	Reverse primer (5'-3')
**METTL3**	CAAGCTGCACTTCAGACGAA-	GCTTGGCGTGTGGTCTTT
**METTL14**	CTACCCATCCTCACTGTCAGTC	GGATGTTCCTGTTTGACCTGAGG
**RBM15**	TCCCACCTTGTGAGTTCTCC	GTCAGCGCCAAGTTTTCTCT
**WTAP**	CTTCCCAAGAAGGTTCGATTGA	TCAGACTCTCTTAGGCCAGTTAC
**VIRMA**	AATCCTGTGGGAAGATCAGC	ACACGTAAGGCAGTGGTAAG
**FTO**	CCAGAACCTGAGGAGAGAATGG	CGATGTCTGTGAGGTCAAACGG
**ALKBH**	CCAGCTATGCTTCAGATCGCCT	GGTTCTCTTCCTTGTCCATCTCC
**YTHDF1**	CTGAGGACGACATCCACCGC	CTTCATCTCGGCCACCCCAC
**YTHDF2**	GGCAGCACTGAAGTTGGG	CTATTGGAAGCCACGATGTTA
**YTHDF3**	CGCCACCCCCGATAAAGCAT	GGTGGAGGCAATGGCTGTGT
**YTHDC2**	CACACTGGAGCCAGAGGCAG	TGGCCTTTGGCCAATCCCAG
**GPX4**	TCCCAGTGAGGCAAGACCGA	GATGCCCTTGCCCTTGGGTT

**GAPDH**	TGACTTCAACAGCGACACCCA	CACCCTGTTGCTGTAGCCAAA

METTL3 mut

A mutated METTL3(DPPW replaced by APPA) construct with disordered enzymatic activity was constructed to evaluated to the critical role of METTL3 mediated m6A methylation on the mRNA stability of GPX4 [9-11].

Reference:

[1].Rittirsch D, Huber-Lang MS, Flierl MA, Ward PA. Immunodesign of experimental sepsis by cecal ligation and puncture. Nat Protoc. 2009;4(1):31-6.

[2].Nascimento DC, Viacava PR, Ferreira RG, Damaceno MA, Piñeros AR, Melo PH, et al. Sepsis expands a CD39+ plasmablast population that promotes immunosuppression via adenosine-mediated inhibition of macrophage antimicrobial activity. Immunity. 2021 Sep 14;54(9):2024-2041.e8

[3].Hubbard WJ, Choudhry M, Schwacha MG, Kerby JD, Rue LW 3rd, Bland KI, Chaudry IH. Cecal ligation and puncture. Shock. 2005 Dec;24 Suppl 1:52-7.

[4].Wang Z, Pu Q, Lin P, Li C, Jiang J, Wu M. Design of Cecal Ligation and Puncture and Intranasal Infection Dual Model of Sepsis-Induced Immunosuppression. J Vis Exp. 2019 Jun 15;(148):10.3791/59386.

[5].Li C, Zhang Y, Liu J, Kang R, Klionsky DJ, Tang D. Mitochondrial DNA stress triggers autophagy-dependent ferroptotic death. Autophagy. 2021 Apr;17(4):948-960.

[6].Dong H, Xia Y, Jin S, Xue C, Wang Y, Hu R, Jiang H. Nrf2 attenuates ferroptosis-mediated IIR-ALI by modulating TERT and SLC7A11. Cell Death Dis. 2021 Oct 29;12(11):1027.

[7].Li Y, Cao Y, Xiao J, Shang J, Tan Q, Ping F, Huang W, Wu F, Zhang H, Zhang X. Inhibitor of apoptosis-stimulating protein of p53 inhibits ferroptosis and alleviates intestinal ischemia/reperfusion-induced acute lung injury. Cell Death Differ. 2020 Sep;27(9):2635-2650.
[8].Liu P, Feng Y, Li H, Chen X, Wang G, Xu S, Li Y, Zhao L. Ferrostatin-1 alleviates lipopolysaccharide-induced acute lung injury via inhibiting ferroptosis. Cell Mol Biol Lett. 2020 Feb 27;25:10
[9].Wang, X., Feng, J., Xue, Y. et al. Structural basis of N6-adenosine methylation by the METTL3–METTL14 complex. Nature 534, 575–578 (2016).
[10].Hao H, Hao S, Chen H, Chen Z, Zhang Y, Wang J, Wang H, Zhang B, Qiu J, Deng F, Guan W. N6-methyladenosine modification and METTL3 modulate enterovirus 71 replication. Nucleic Acids Res. 2019 Jan 10;47(1):362-374.
[11].Xia H, Zhong C, Wu X, Chen J, Tao B, Xia X, Shi M, Zhu Z, Trudeau VL, Hu W. Mettl3 Mutation Disrupts Gamete Maturation and Reduces Fertility in Zebrafish. Genetics. 2018 Feb;208(2):729-743.

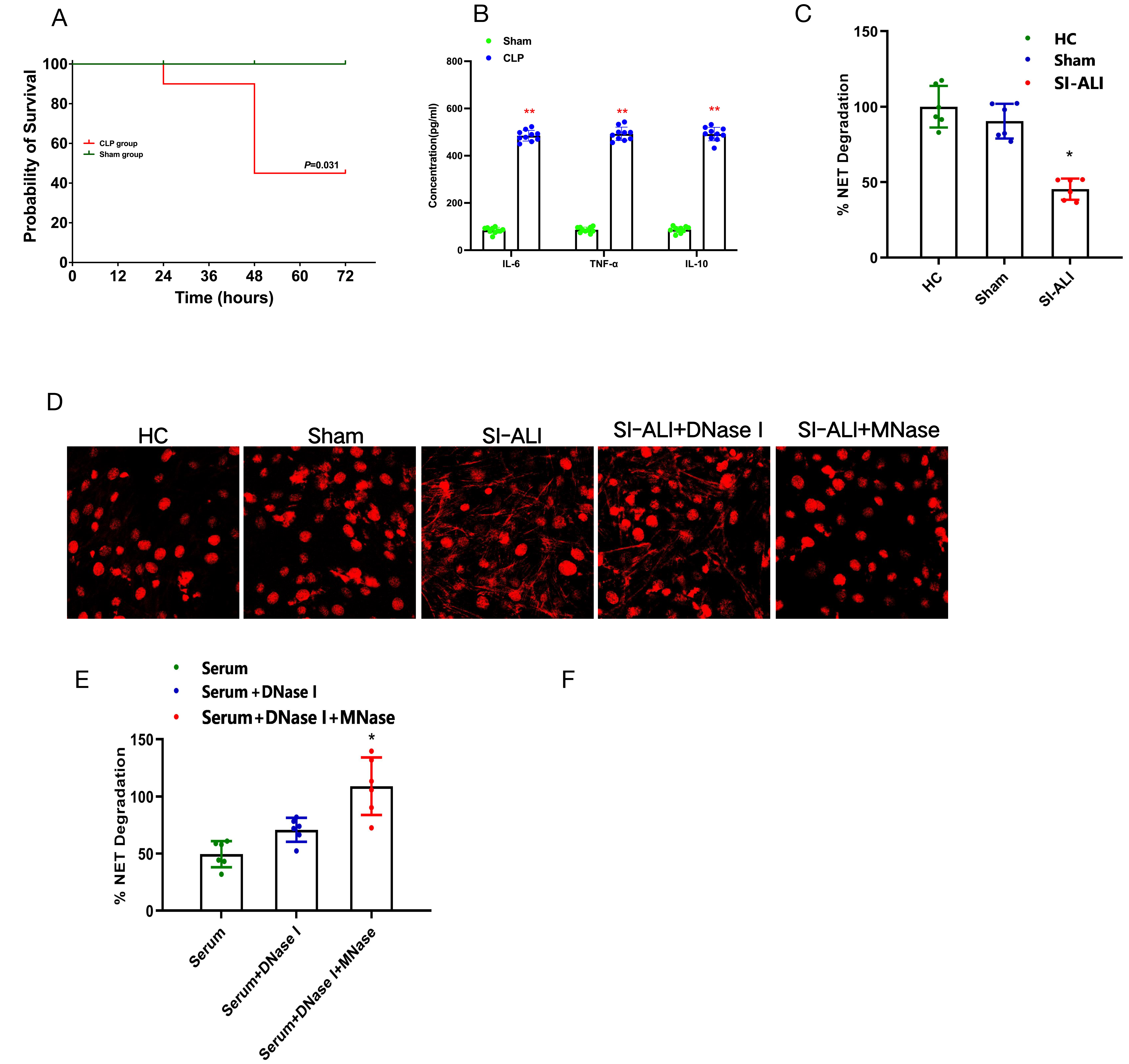

Supplementary Figure 1

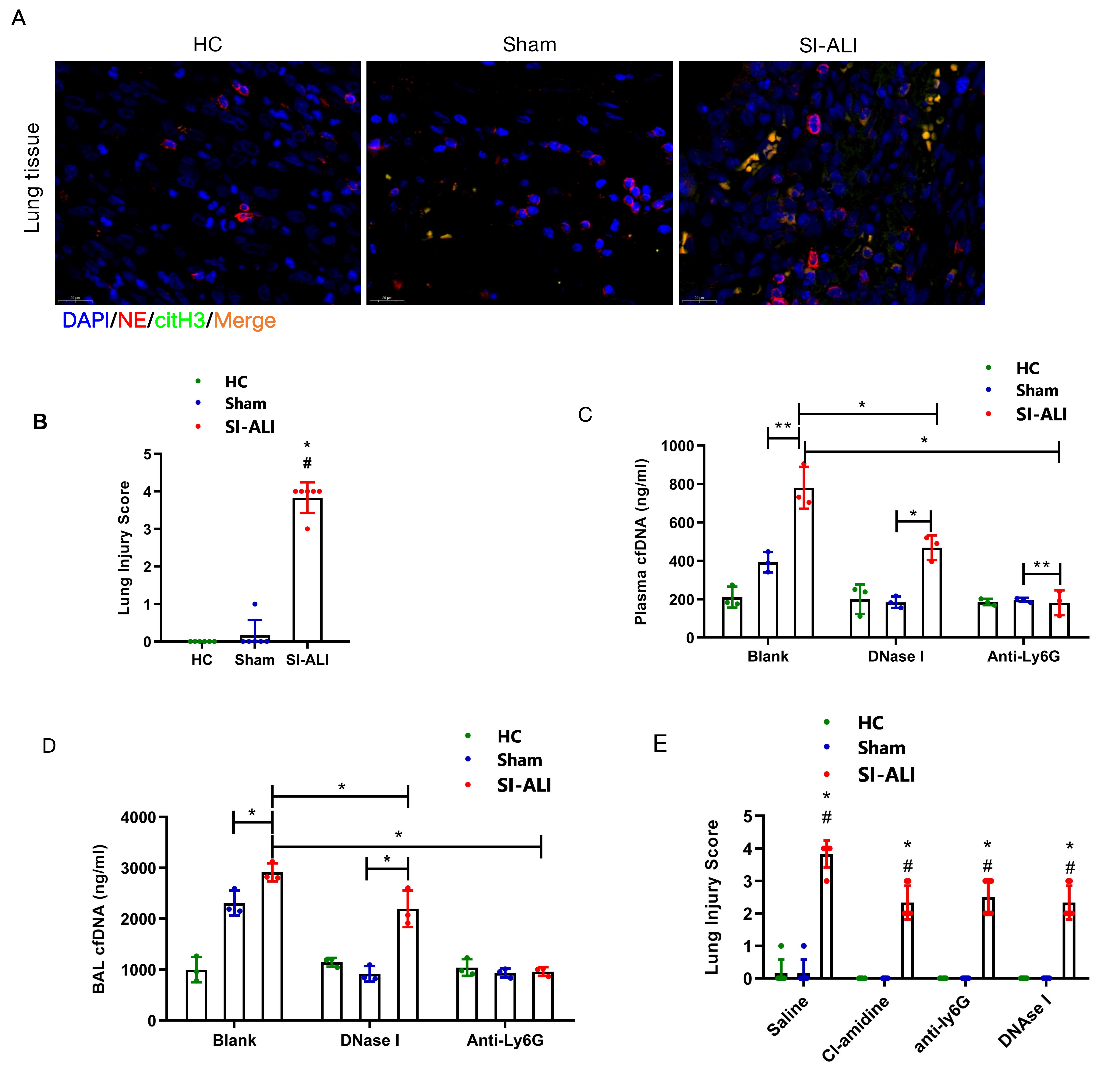

Supplementary Figure 2

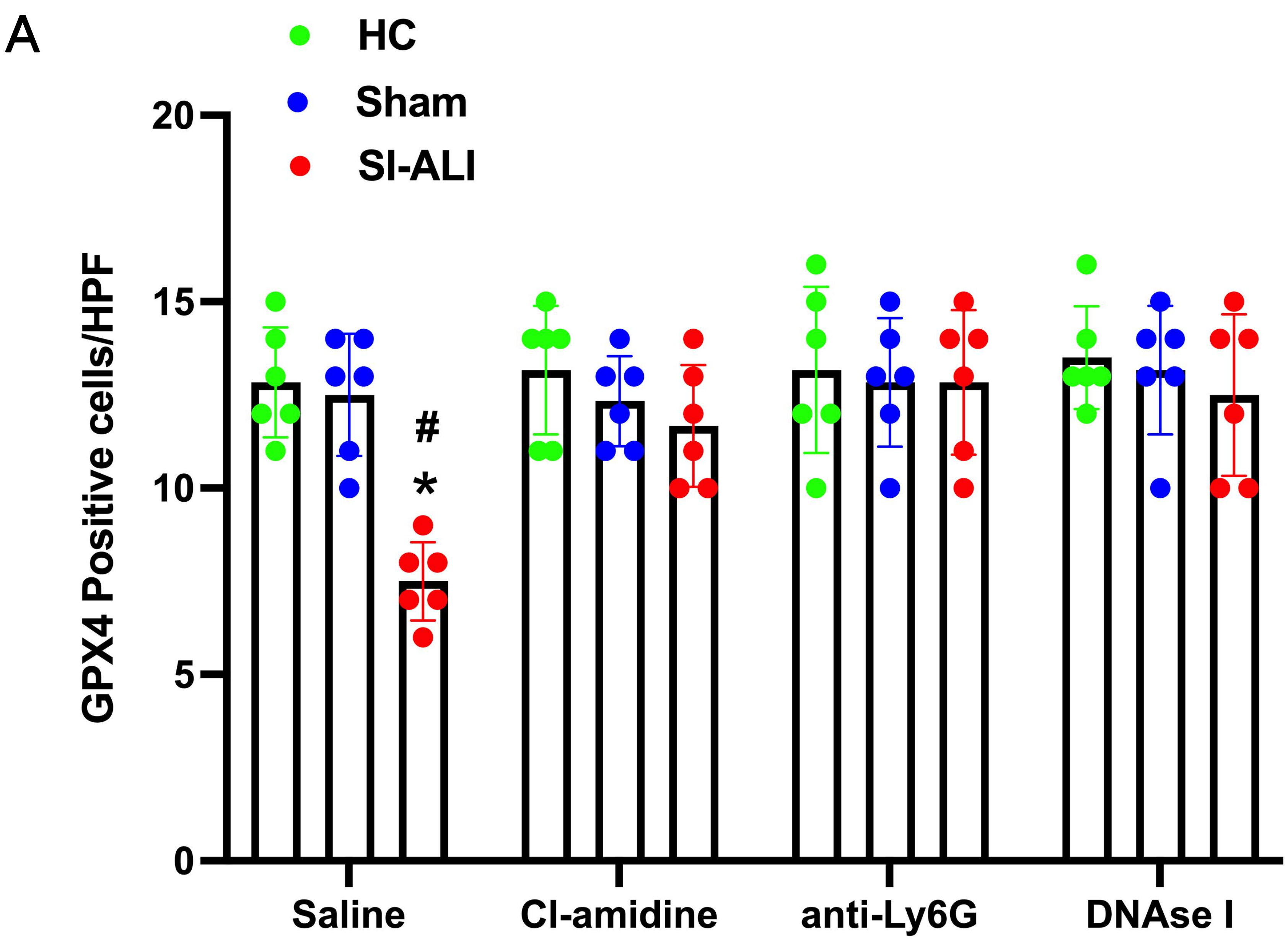

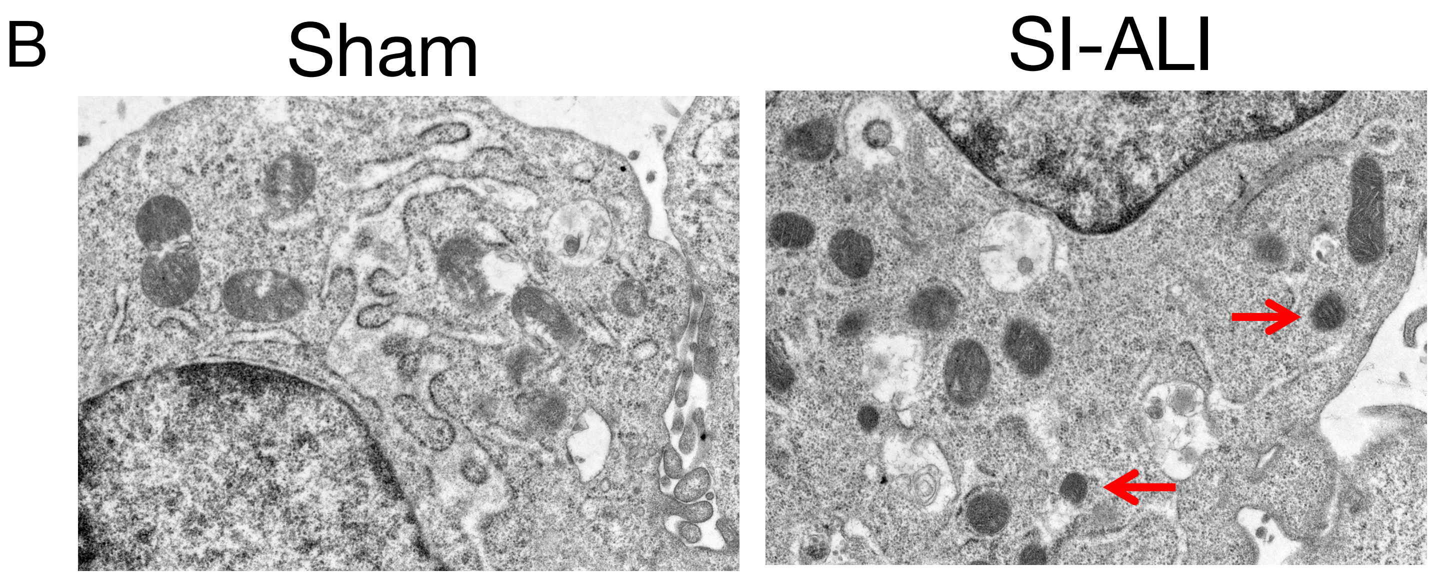

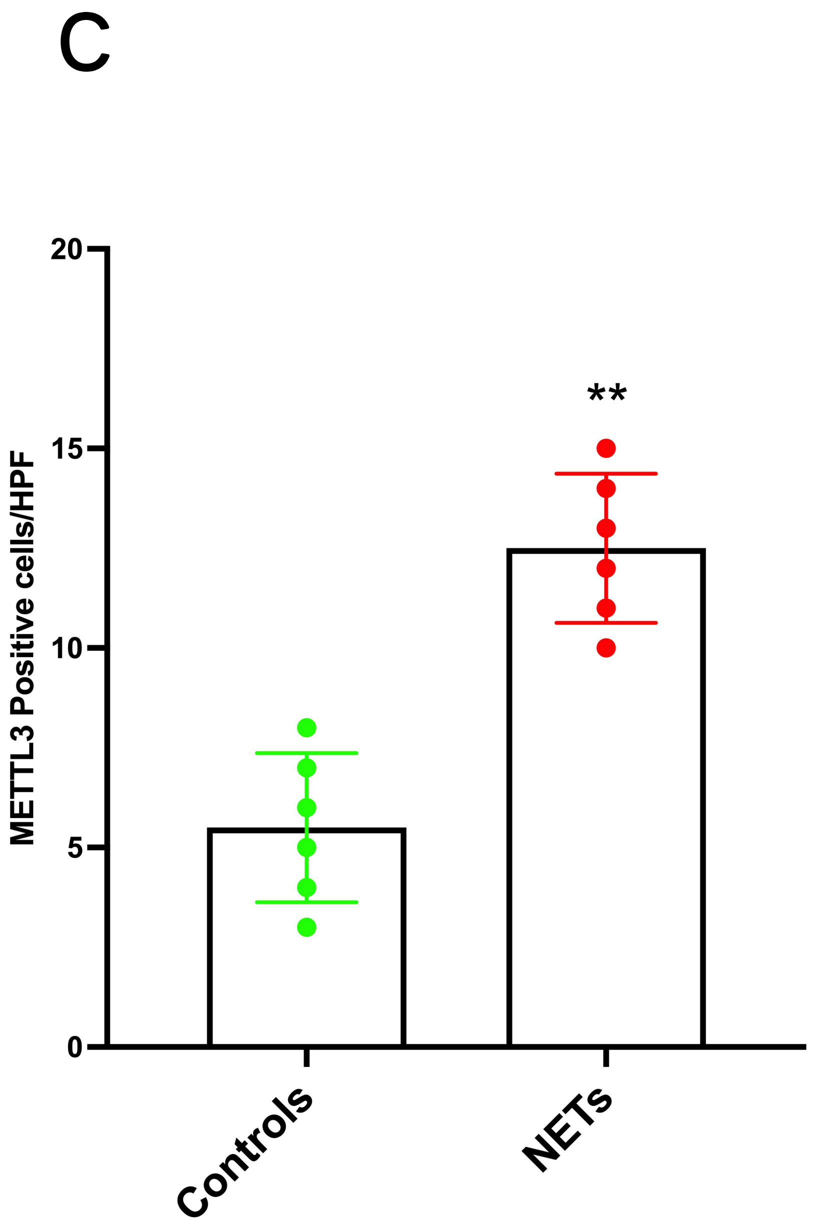

Supplementary Figure 3

A

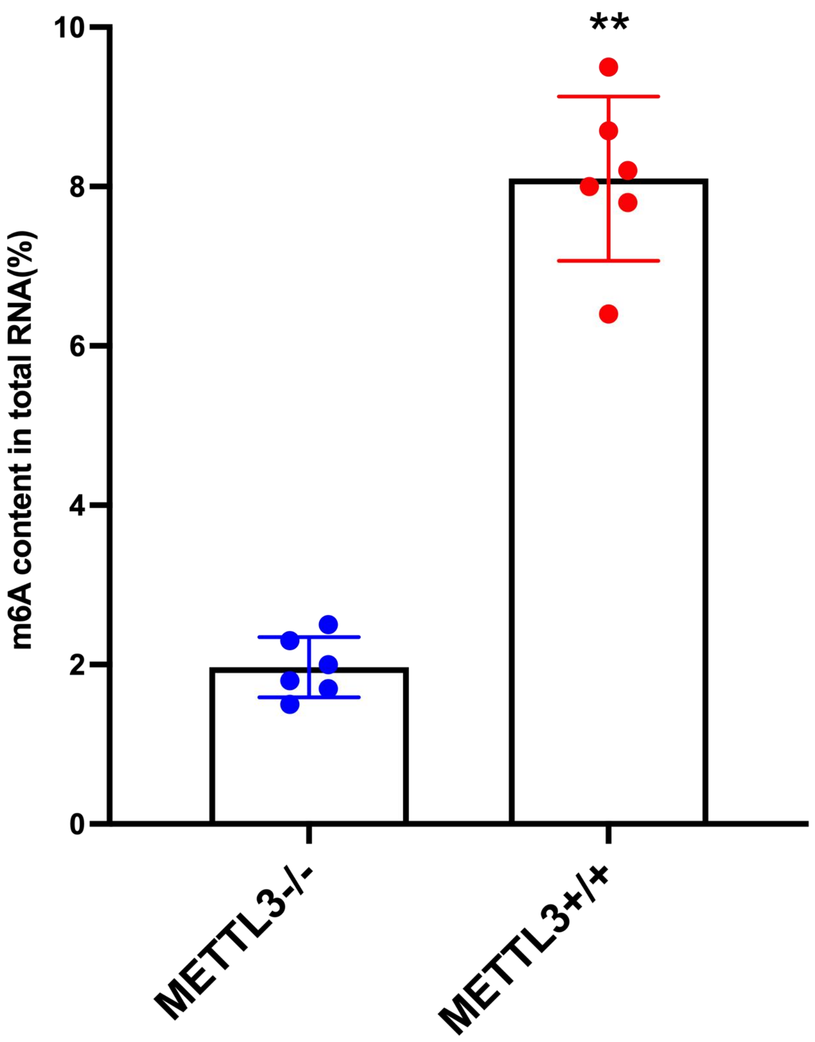

Supplementary Figure 4

A

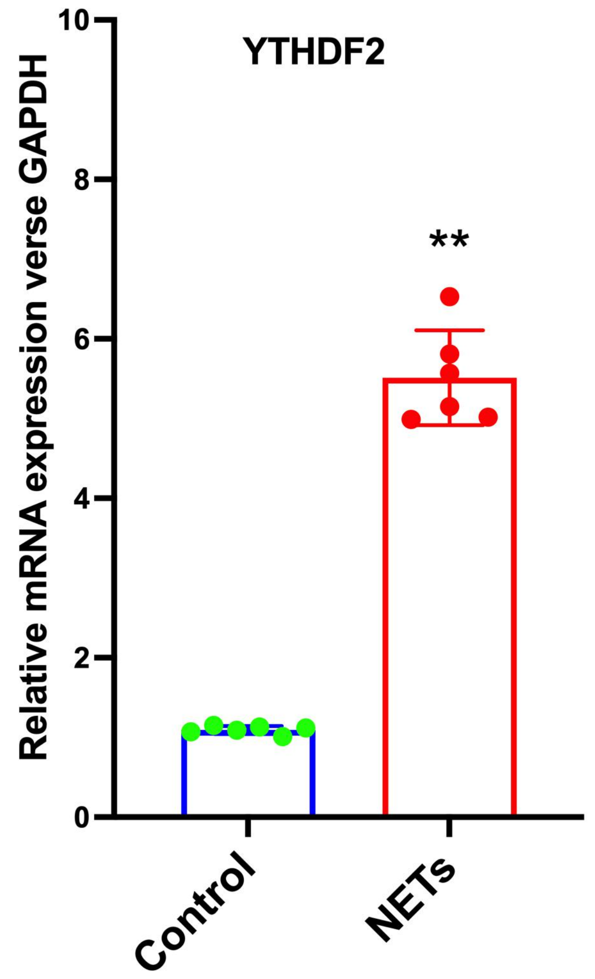

B

Position

#	Position	Sequence context	Structural context	Local structure visualization	Score(binary)	Score(knn)	Score(spectrum)	Score(combined)	Decision
1	193	AUGCA CGAGU UUUCC<br>GCCAA GGACA UCGAC<br>GGGCA CAUGG UUAAC	N/A	N/A	0.846	0.708	0.808	0.824	m6A site (**Very high** confidence)
2	647	GAGCC CCUGG UGAUA<br>GAGAA GGACC UGCCC<br>CACUA UUUCU AGCUC	N/A	N/A	0.808	0.474	0.822	0.797	m6A site (**Very high** confidence)
3	766	UGCAA ACCUG CUGGU<br>GGGGC AGACC CGAAA<br>AUCCA GCGUG CACCC	N/A	N/A	0.752	0.240	0.789	0.741	m6A site (**Very high** confidence)

C

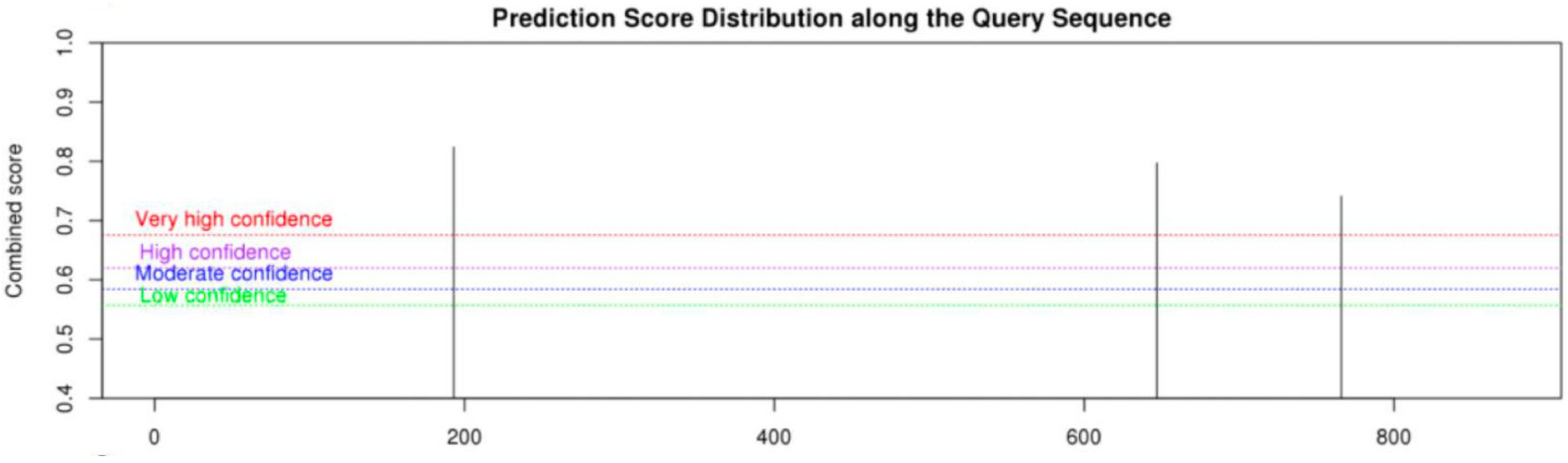

Supplementary Figure 5

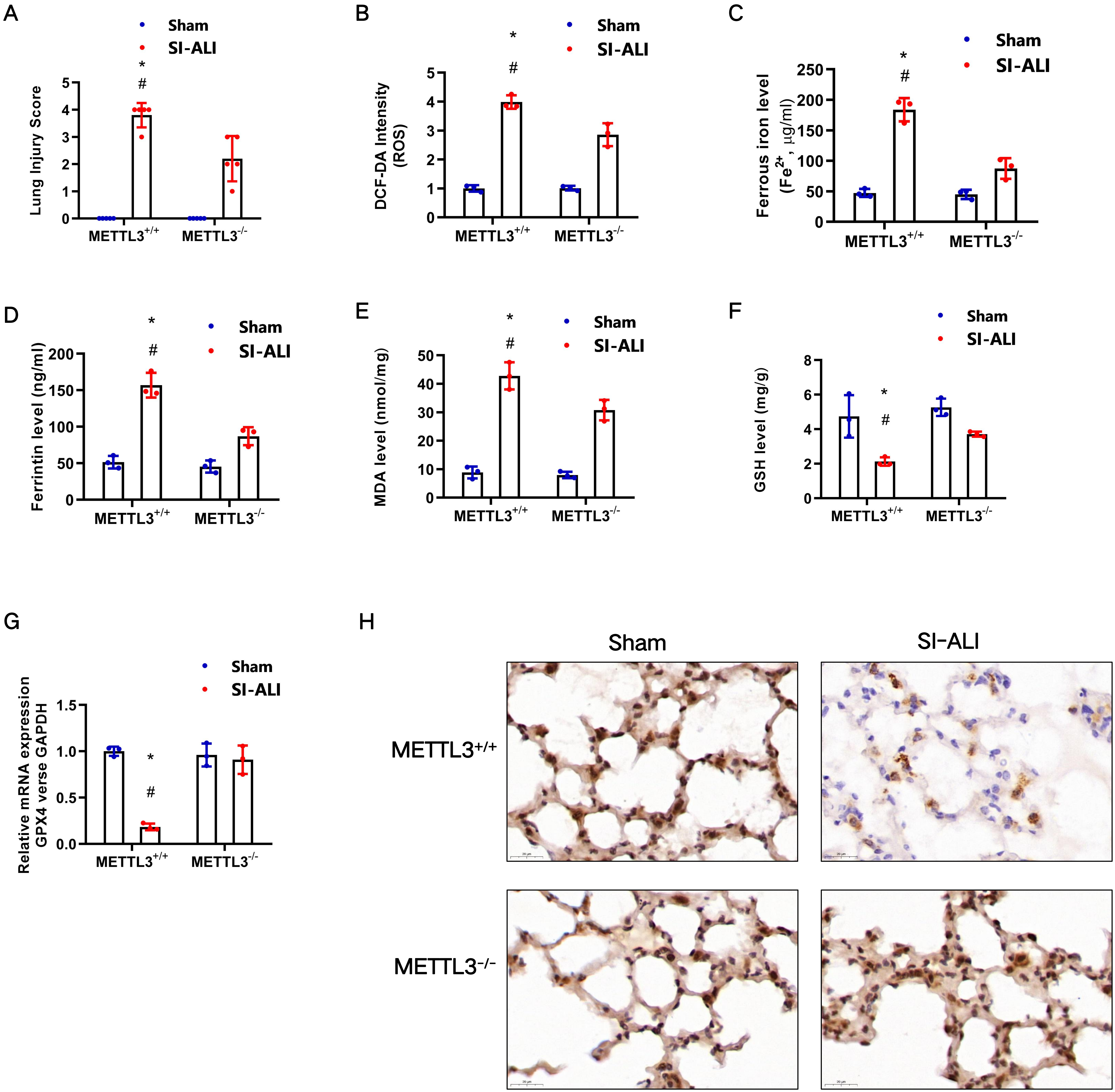

Supplementary Figure 6

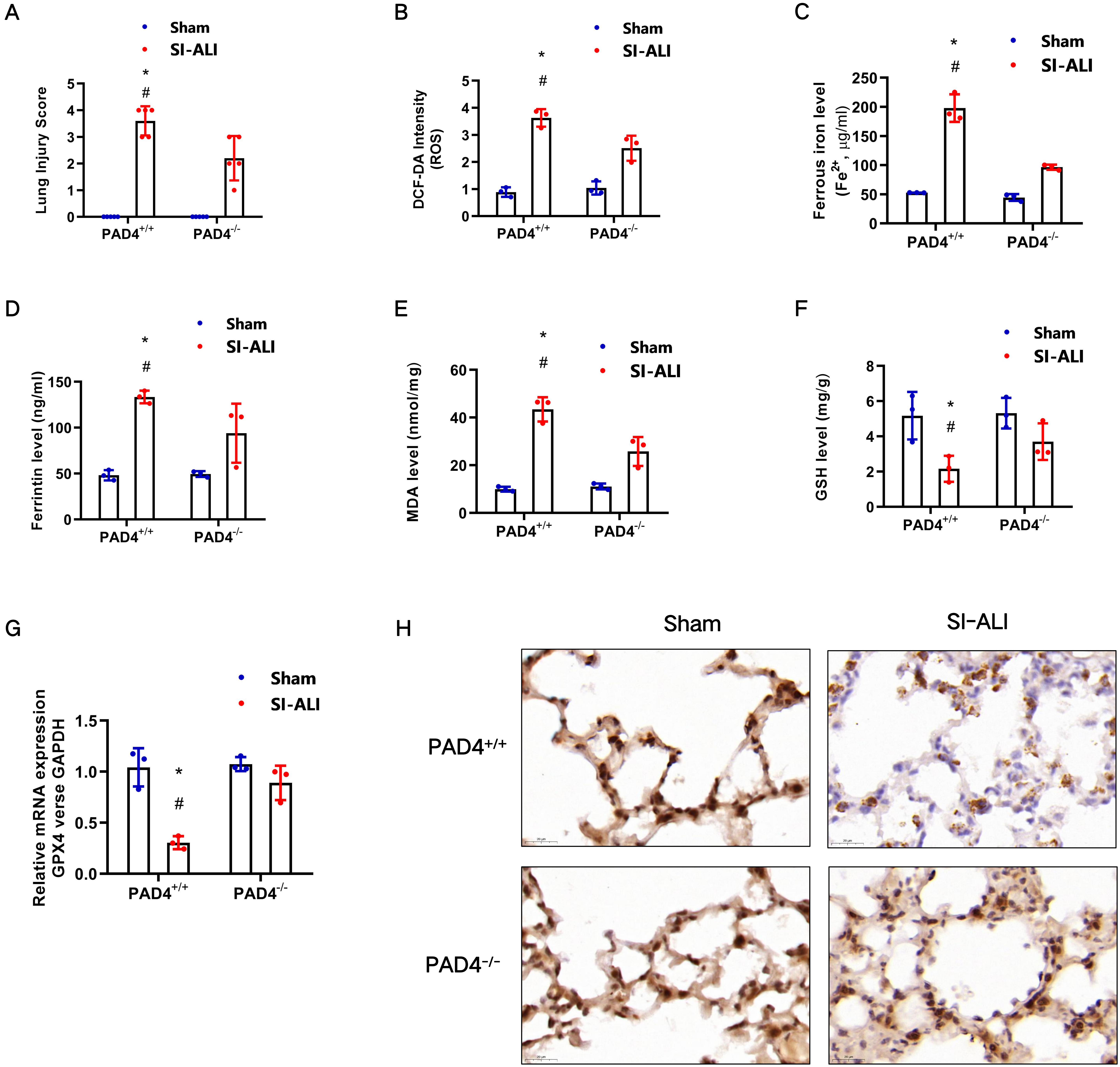

Supplementary Figure 7

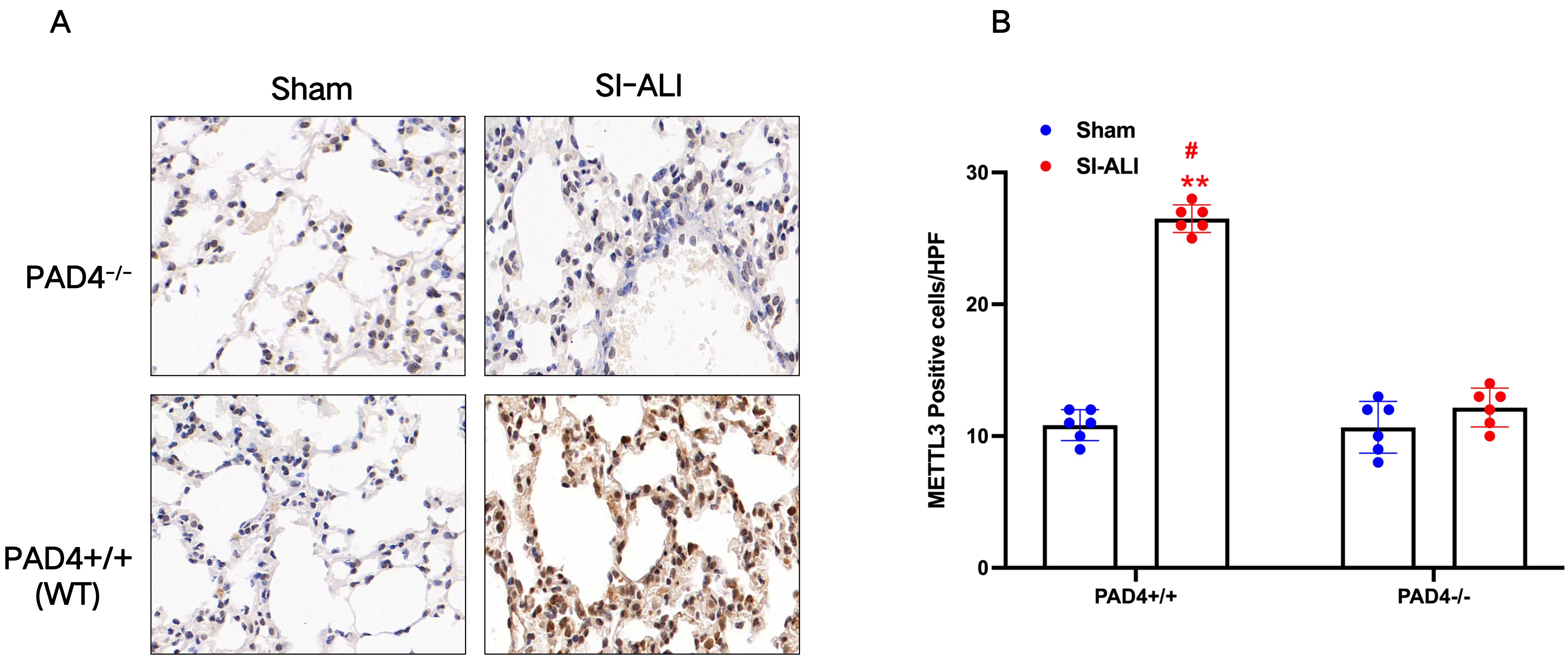

Supplementary Figure 8

Supplementary Table. 1 Baseline of characteristics of healthy and patients enrolled in the group

	Healthy Control (N=20)	Sepsis (N=24)	Sepsis ARDS (N=22)
**Gender (male, %)**	12(60%)	12(50%)	11(50%)
**Ages (years), mean±SD**	44.3±13.6	44.4±12.8	45.8±13.2
**BMI (kg/m^2), mean±SD**	27.6±6.3	27.4±6.4	27.3±6.3
**Neutrophils (10^9/L)**	3.2±1.8	5.0±1.4*	6.8±3.2*$^\#$
**Monocytes (10^9/L)**	0.7±0.2	0.7±0.3	0.8±0.2
**Lymphocytes (10^9/L)**	1.9±0.3	1.8±0.5	1.7±0.4
**Platelets (10^9/L)**	232.9±38.4	343.7±105.4*	392.5±176.4*$^\#$
**Hemoglobin (g/L)**	143.2±12.5	122.3±27.5*	103.6±22.1*$^\#$
**ESR (mm/h)**	8.1±2.1	28.3±3.4*	48.7±21.5*$^\#$
**CRP (mg/L)**	4.3±0.5	75.1±30.2*	128.3±43.2*$^\#$
**Albumin(g/L)**	46.2±2.5	36.2±6.1*	31.5±6.8*$^\#$
**PT(s)**	11.1±1.0	10.5±1.4	10.8±1.5
**APTT(s)**	32.4±3.2	33.4±5.4	35.4±4.3
**D-dimer(ng/ml)**	90±67.4	195.4±93.5*	328.5±106.2*$^\#$
**Fibrinogen(g/L)**	2.8±0.4	4.6±1.0*	7.2±1.1*$^\#$
**PaO2/FiO2**	410±26	330±30*	260±25*$^\#$
**Diagnosis**			
**Intestinal obstruction**	NA	12(50%)	11(50%)
**Trauma Injury**	NA	12(50%)	11(50%)
**Surgery Type**			
**Intestinal resection(%)**	NA	12(50%)	11(50%)
**Hepatectomy(%)**	NA	12(50%)	11(50%)
**Mechanically Ventilation**(%)	NA	6(25%)	22(100%)$^\#$
**SOFA score(IQR)**	NA	8(6,9)	11(8,13)$^\#$

Abbreviations: BMI: Body Mass Index, ESR: Erythrocyte Sedimentation Rate, IQR: Interquartile range, CRP: C reactive protein,SOFA:Sequential Organ Failure Assessment

Data are expressed by percentage or median (interquartile range [IQR]), mean ± standard deviation [SD], *P < 0.01 versus controls; $^\#$P < 0.01 versus sepsis patients
